# Association is not causation: treatment effects cannot be estimated from observational data in heart failure

**DOI:** 10.1093/eurheartj/ehy407

**Published:** 2018-08-01

**Authors:** Christopher J Rush, Ross T Campbell, Pardeep S Jhund, Mark C Petrie, John J V McMurray

**Affiliations:** British Heart Foundation Cardiovascular Research Centre, Institute of Cardiovascular and Medical Sciences, University of Glasgow, 126 University Place, Glasgow, UK

**Keywords:** Heart failure, Pharmacotherapy, Associations, Observational studies, Randomized controlled trials

## Abstract

**Aims:**

Treatment ‘effects’ are often inferred from non-randomized and observational studies. These studies have inherent biases and limitations, which may make therapeutic inferences based on their results unreliable. We compared the conflicting findings of these studies to those of prospective randomized controlled trials (RCTs) in relation to pharmacological treatments for heart failure (HF).

**Methods and results:**

We searched Medline and Embase to identify studies of the association between non-randomized drug therapy and all-cause mortality in patients with HF until 31 December 2017. The treatments of interest were: angiotensin-converting enzyme inhibitors, angiotensin receptor blockers, beta-blockers, mineralocorticoid receptor antagonists (MRAs), statins, and digoxin. We compared the findings of these observational studies with those of relevant RCTs. We identified 92 publications, reporting 94 non-randomized studies, describing 158 estimates of the ‘effect’ of the six treatments of interest on all-cause mortality, i.e. some studies examined more than one treatment and/or HF phenotype. These six treatments had been tested in 25 RCTs. For example, two pivotal RCTs showed that MRAs reduced mortality in patients with HF with reduced ejection fraction. However, only one of 12 non-randomized studies found that MRAs were of benefit, with 10 finding a neutral effect, and one a harmful effect.

**Conclusion:**

This comprehensive comparison of studies of non-randomized data with the findings of RCTs in HF shows that it is not possible to make reliable therapeutic inferences from observational associations. While trials undoubtedly leave gaps in evidence and enrol selected participants, they clearly remain the best guide to the treatment of patients.

## Introduction

**Figure 1:**
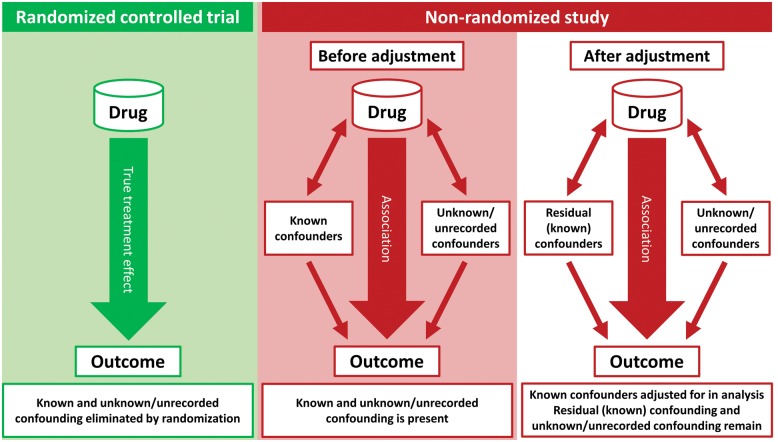
Confounding in non-randomized studies.

**Figure 2:**
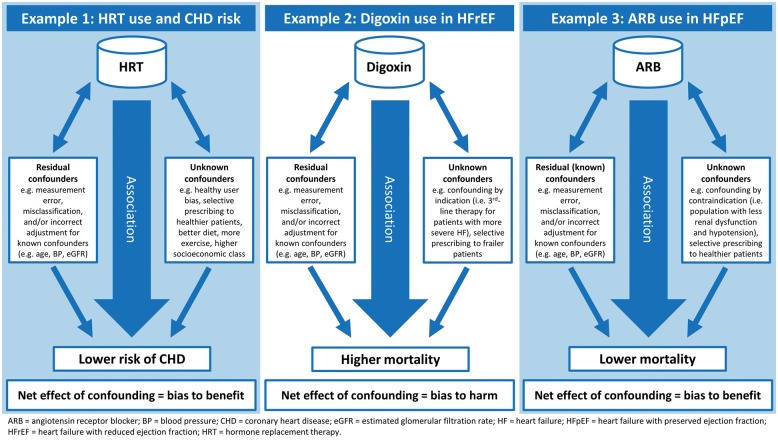
Examples of confounding in non-randomized studies.

Randomized controlled trials (RCTs) are widely acknowledged to be the gold standard test of whether or not a drug is beneficial.^1–4^ Although the biases and limitations of non-randomized, observational studies have been recognized for decades (*Figure 1*), studies of this type purporting to describe the effects of treatment continue to be published, even in high-impact journals.^5–10^ Indeed, the ‘comparative effectiveness’ and ‘big data’ movements have given non-randomized studies a new respectability in some peoples’ eyes.^11–13^ Advocates point to the use of more sophisticated analytical techniques than in the past and increasingly larger ‘real-world’ datasets.^14–17^ If the findings of observational studies could validly determine the effect of treatments, such information would clearly be of considerable value. On the other hand, if such analyses are inherently flawed they serve only to cause confusion, e.g. the association between hormone replacement therapy and decreased risk of coronary heart disease (CHD)^18^^,^^19^ (*Figure 2*), and maybe worse, e.g. lead to discontinuation of effective therapy by physicians or patients misled by the findings.^20^

There is a particularly strong evidence base for pharmacological treatments in heart failure (HF), making it an appropriate condition in which to compare treatment effects established in RCTs with those reported in non-randomized studies. We have, therefore, compared the conflicting results of non-randomized studies of the ‘effect’ of pharmacological treatments with those of RCTs using the same therapies for HF. Although many publications of this type have used the word ‘effect’, more correctly they have actually described associations between treatments and outcomes.

## Methods

### Search strategy and eligibility criteria

We conducted a comprehensive search of the electronic databases Medline and Embase to identify observational studies examining the association between non-randomized drug therapy and all-cause mortality in patients with HF. The drugs of interest were those included in all major HF guidelines: angiotensin-converting enzyme inhibitors (ACEIs), angiotensin receptor blockers (ARBs), beta-blockers, mineralocorticoid receptor antagonists (MRAs), statins (3-hydroxy-3-methyl-glutaryl-coenzyme A reductase inhibitors), and digoxin, where the effect on all-cause mortality had been tested in at least one large RCT.^21^^,^^22^ The term ‘heart failure’ was searched in title and keywords relating to outcome data and pharmacotherapy were searched in title or abstract to retrieve all potentially relevant articles (see Supplementary material online, *Figures S1*–*S5*). The search, updated until 31 December 2017, was limited to studies of adults, published in the English language, with more than 100 participants in both the study drug and control groups, with a minimum follow-up period of six months. Studies of patients with left ventricular systolic dysfunction and/or HF after myocardial infarction were not included. We also excluded studies describing only subgroups of patients with HF, e.g. those with HF and chronic kidney disease, HF and diabetes etc. Bibliographies of meta-analyses, guidelines, reviews, and manuscripts identified through the search strategy were also hand-searched for additional eligible studies. The review was conducted according to the Preferred Reporting Items for Systematic reviews and Meta-Analyses (PRISMA) guidelines.^23^

Non-randomized studies were considered for inclusion in this review if the following requirements were met:

1. Inclusion of patients with HF
2. Report of the ‘effect’ of the drug of interest on all-cause mortality
3. Estimate of treatment ‘effect’ provided as a multivariate-adjusted hazard ratio (HR), risk ratio/relative risk, or odds ratio

### Data extraction, synthesis, and risk of bias

Data from the manuscripts identified through the search criteria were abstracted and tabulated by one reviewer (C.J.R.). The data were independently verified by a second reviewer (R.T.C.), with a third reviewer (J.J.M.) resolving any discrepancies. The articles retrieved were categorized according to HF phenotype, based on ejection fraction (EF), and drug class for comparison with the relevant randomized trials. For studies that reported more than one multivariable-adjusted ‘effect’ estimate, the estimate which had been adjusted for most confounders was used. A two-tailed *P*-value of 0.05 was considered significant.

The quality of each study was assessed with the Cochrane Collaboration Risk of Bias tool for RCTs and the Risk of Bias Assessment tool for Non-randomized Studies (RoBANS) tool for observational studies (see Supplementary material online, *Tables S1 and S2*).^24^^,^^25^ Studies judged as having a low risk of bias have been presented separately from those with a high or unclear risk of bias in the Supplementary material online, *Figures S6–S19*.

## Results

We identified 92 publications reporting 94 non-randomized studies.^26–117^ Together, these described 158 estimates of the ‘effect’ of the six treatments of interest on all-cause mortality. These six treatments had been tested in 25 RCTs.^118–147^ The results of our analyses are summarized in *Table 1* and described in detail in *Tables 2–6*. The forest plots in the Supplementary material online, *Figures S6–S19* illustrate the treatment effects/association between treatment and outcomes in the trials and observational studies, respectively, reported in *Tables 2–6* and include a quality assessment of these trials/studies.

**Table 1:** Summary of the concordance between the effect of treatment on mortality in randomized controlled trials and the association between non-randomized use of the same treatments and mortality in observational studies in HF

Treatment	Randomized controlled trials	Observational studies		
		Benefit	Neutral	Harm
HFrEF				
ACEI/ARB	Benefit	5	2	0
Beta-blocker	Benefit	16	2	0
MRA	Benefit	1	10	1
Statin	Neutral	14	3	0
Digoxin	Neutral	1	4	5
HFpEF				
ACEI/ARB	Neutral	5	7	0
Beta-blocker	Neutral	9	4	0
MRA	Neutral	1	2	0
Statin	—	—	—	—
Digoxin	Neutral	1	3	0
Mixed/unspecified HF phenotype				
ACEI/ARB	Neutral	8	2	0
Beta-blocker	Neutral	17	2	0
MRA	—	2	3	0
Statin	Neutral	11	1	0
Digoxin	Neutral	2	7	7

ACEI, angiotensin-converting enzyme inhibitor; ARB, angiotensin receptor blocker; HF, heart failure; HFpEF, heart failure with preserved ejection fraction; HFrEF, heart failure with reduced ejection fraction; MRA, mineralocorticoid receptor antagonist.

**Table 2:** All-cause mortality in randomized and non-randomized ACEI/ARB HF studies

First author, country, year of publication (study name)	Study design	Study period	Region	Mean follow -up (months)	Patients (*n*)	Study (*n*)	Control (*n*)	All-cause mortality—unadjusted HR (95% CI)	All-cause mortality—adjusted HR (95% CI)
HFrEF (ACEI)									
Randomized controlled trials—beneficial treatment effect									
SOLVD Investigators, USA, 1991 (SOLVD-Treatment)^118^	RCT	1986–1989	USA, Canada, Belgium	41	2569	1285	1284	RR: 0.84 (0.74–0.95; *P* < 0.004)	—
Jong, Canada, 2003 (X-SOLVD Overall)^119^	RCT	1986–1990	USA, Canada, Belgium	134–145^a^	6797	3396	3401	0.90 (0.84–0.95; *P* < 0.0003)	—
Jong, Canada, 2003 (X-SOLVD-Prevention)^119^	RCT	1986–1990	USA, Canada, Belgium	134^a^	4228	2111	2117	0.86 (0.79–0.93; *P* < 0.001)	—
Randomized controlled trials—neutral treatment effect									
SOLVD Investigators, USA, 1992 (SOLVD-Prevention)^120^	RCT	1986–1990	USA, Canada, Belgium	37	4228	2111	2117	RR: 0.92 (0.79–1.08; *P* < 0.30)	—
Jong, Canada, 2003 (X-SOLVD-Treatment)^119^	RCT	1986–1990	USA, Canada, Belgium	145^a^	2569	1285	1284	0.93 (0.85–1.01; *P* < 0.01)	—
Observational studies—beneficial treatment effect									
Masoudi, USA, 2004 (NHC)^26^	Retrospective cohort study (≥65 years)	1998–1999, 2000–2001	USA	12	17 456	12 069	13 600	RR: 0.78 (0.75–0.81; *P* < 0.0001)	RR: 0.86 (0.82–0.90)
HFrEF (ARB)									
Randomized controlled trials—neutral treatment effect									
Granger, USA, 2003 (CHARM-Alternative)^121^	RCT	1999–2001	Multiregional	34^a^	2028	1013	1015	0.87 (0.74–1.03; *P* < 0.11)	0.83 (0.70–0.99; *P* < 0.033)
HFrEF (ACEI + ARB)									
Observational studies—beneficial treatment effect									
Sanam, USA, 2016 (Alabama HF Project)^27^	Retrospective cohort study (PSM) (≥65 years)	1998–2001	USA	12	954	477	477	—	0.77 (0.62–0.96; *P* < 0.020)
Liu, China, 2014^28^	Prospective cohort study	2005–2010	China	52^a^	2154	1421	733	—	0.43 (0.33–0.57; *P* < 0.001)
Lund, Sweden, 2012 (Swedish HF Registry)^29^	Registry (PSM)	2000–2011	Sweden	12	4010	2005	2005	—	0.80 (0.74–0.86; *P* < 0.001)
Masoudi, USA, 2004 (NHC)^26^	Retrospective cohort study (≥65 years)	1998–1999, 2000–2001	USA	12	17 456	13 600	3856	—	RR: 0.83 (0.79–0.88)
** **Observational studies—neutral treatment effect									
Ushigome, Japan, 2015 (1. CHART-1)^30^	Prospective cohort study	2000–2005	Japan	36	543	385	158	—	0.67 (0.40–1.12; *P* < 0.128)
Ushigome, Japan, 2015 (2. CHART-2)^30^	Prospective cohort study	2006–2010	Japan	36	1360	1061	299	—	0.83 (0.60–1.15; *P* < 0.252)
HFpEF (ACEI)									
Randomized controlled trials—neutral treatment effect									
Cleland, UK, 2006 (PEP-CHF)^122^	RCT (≥70 years)	2000–2003	Multiregional	26	850	424	426	1.09 (0.75–1.58; *P* < 0.665)	—
Observational studies—beneficial treatment effect									
Gomez-Soto, Spain, 2010^31^	Prospective cohort study (propensity score adjusted)	2001–2005	Spain	30^a^	1120	255	865	RR: 0.34 (0.23–0.46; *P* < 0.001)	0.67 (0.52–0.71)
Shah, USA, 2008 (NHC)^32^	Retrospective cohort study (≥65 years)	1998–1999, 2000–2001	USA	36	13 533	6413	7120	—	RR: 0.93 (0.89–0.98)
Tribouilloy, France, 2008^33^	Prospective cohort study (PSM)	2000	France	60	240	120	120	0.61 (0.43–0.87; *P* < 0.006)	0.58 (0.40–0.82; *P* < 0.002)
Grigorian Shamagian, Spain, 2006^34^	Prospective cohort study	1991–2002	Spain	31	416	210	206	0.56 (0.40–0.79; *P* < 0.001)	0.63 (0.44–0.90; *P* < 0.012)
Observational studies—neutral treatment effect									
Mujib, USA, 2013 (OPTIMIZE-HF)^35^	Registry (PSM) (≥65 years)	2003–2004	USA	29^a^	2674	1337	1337	—	0.96 (0.88–1.05; *P* < 0.373)
Dauterman, USA, 2001 (Medicare)^36^	Retrospective cohort study (≥65 years)	1993–1994, 1996	USA	12	430	206	224	—	1.15 (0.79–1.67; *P* < 0.46)
Philbin, USA, 2000 (MISCHF)^37^	Registry	1995, 1996–1997	USA	6	302	137	165	OR: 0.72 (0.38–1.39)	OR: 0.61 (0.30–1.25)
Philbin, USA, 1997 (MISCHF)^38^	Registry	1995	USA	6	350	190	160	—	OR: 0.63 (*P* < 0.15–95% CI not reported)
HFpEF (ARB)									
Randomized controlled trials—neutral treatment effect									
Massie, USA, 2008 (I-PRESERVE)^123^	RCT	2002–2005	Multiregional	50	4128	2067	2061	1.00 (0.88–1.14; *P* < 0.98)	—
Yusuf, Canada, 2003 (CHARM-Preserved)^124^	RCT	1999–2000	Multiregional	37^a^	3023	1514	1509	1.02 (0.85–1.22; *P* < 0.836)	—
Observational studies—neutral treatment effect									
Patel, USA, 2012 (OPTIMIZE-HF)^39^	Registry (PSM) (≥65 years)	2003–2004	USA	72	592	296	296	0.93 (0.76–1.14; *P* < 0.509)	—
HFpEF (ACEI + ARB)									
Observational studies—beneficial treatment effect									
Lund, Sweden, 2012 (Swedish HF Registry)^29^	Registry (PSM)	2000–2011	Sweden	12	6658	3329	3329	—	0.91 (0.85–0.98; *P* < 0.008)
** **Observational studies—neutral treatment effect									
Ushigome, Japan, 2015 (1. CHART-1)^30^	Prospective cohort study	2000–2005	Japan	36	463	304	159	—	0.86 (0.51–1.47; *P* < 0.592)
Ushigome, Japan, 2015 (2. CHART-2)^30^	Prospective cohort study	2006–2010	Japan	36	2316	1619	697	—	1.01 (0.77–1.32; *P* < 0.924)
Mixed/unspecified HF phenotype (ACEI)									
** **Randomized controlled trials—beneficial treatment effect									
Cohn, USA, 1991 (V-HeFT-II)^125^	RCT	1986–1990	USA	24	804	403	401 (H-ISDN)	RR: 0.72 (*P* < 0.016–95% CI not reported)	—
CONSENSUS Trial Study Group, Sweden, 1987 (CONSENSUS)^126^	RCT	1985–1986	Sweden, Norway, Finland	12	245	127	126	RR: 0.69 (*P* < 0.001–95% CI not reported)	—
** **Observational studies—beneficial treatment effect									
Keyhan, Canada, 2007 (1. female cohort)^40^	Retrospective cohort study (≥65 years)	1998–2003	Canada	12	14 693	9801	4892	0.75 (0.71–0.78)	0.80 (0.76–0.85)
Keyhan, Canada, 2007 (2. male cohort)^40^	Retrospective cohort study (≥65 years)	1998–2003	Canada	12	13 144	9419	3725	0.62 (0.59–0.65)	0.71 (0.67–0.75)
Tandon, Canada, 2004 (75% HFrEF, 25% HFpEF)^41^	Prospective cohort study	1989–2001	Canada	32^a^	1041	878	163	—	OR: 0.60 (0.39–0.91)
Pedone, Italy, 2004 (GIFA)^42^	Prospective cohort study (≥65 years)	1998	Italy	10	818	550	268	0.56 (0.41–0.78)	0.60 (0.42–0.88)
Ahmed, USA, 2003 (Medicare)^43^	Retrospective cohort study (PSM)	1994	USA	36	1090	528	562	0.77 (0.66–0.91)	0.81 (0.69–0.97)
Sin, Canada, 2002 (19% HFrEF, 36% HFpEF, 45% unknown)^44^	Retrospective cohort study (≥65 years) (propensity score adjusted)	1994–1998	Canada	21^a^	11 942	4908	7034	—	0.59 (0.55–0.62)
Mixed/unspecified HF phenotype (ARB)									
** **Randomized controlled trials—neutral treatment effect									
Pfeffer, USA, 2003 (CHARM Overall Programme) (60% HFrEF, 40% HFpEF)^127^	RCT	1999–2001	Multiregional	40^a^	7599	3803	3796	0.91 (0.83–1.00; *P* < 0.055)	0.90 (0.82–0.99; *P* < 0.032)
Mixed/unspecified HF phenotype (ACEI + ARB)									
** **Observational studies—beneficial treatment effect									
Gastelurrutia, Spain, 2012 (75% HFrEF, 25% HFrEF)^45^	Prospective cohort study	2001–2008	Spain	44^a^	960	846	114	—	0.52 (0.39–0.69; *P* < 0.001)
Teng, Australia, 2010 (WAHMD) (24% HFrEF, 30% HFpEF, 46% unknown)^46^	Retrospective cohort study	1996–2006	Australia	12	944	701	243	—	0.71 (0.57–0.89; *P* < 0.003)
** **Observational studies—neutral treatment effect									
Ushigome, Japan, 2015 (1. CHART-1) (54% HFrEF, 46% HFpEF)^30^	Prospective cohort study	2000–2005	Japan	36	1006	689	317	—	0.79 (0.55–1.14; *P* < 0.208)
Ushigome, Japan, 2015 (2. CHART-2) (37% HFrEF, 63% HFpEF)^30^	Prospective cohort study	2006–2010	Japan	36	3676	2677	999	—	0.94 (0.76–1.15; *P* < 0.534)

a Median.

—, Not reported; ACEI, angiotensin-converting enzyme inhibitor; ARB, angiotensin receptor blocker; CHARM, Candesartan in Heart Failure Assessment of Reduction in Mortality and Morbidity; CHART, Chronic Heart Failure Analysis and Registry in the Tohoku district; CI, confidence interval; CONSENSUS, Cooperative North Scandinavian Enalapril Survival Study; GIFA, Gruppo Italiano di Farmacovigilanza nell'Anziano; HF, heart failure; HFpEF, heart failure with preserved ejection fraction; HFrEF, heart failure with reduced ejection fraction; H-ISDN, hydralazine-isosorbide dinitrate; HR, hazard ratio; I-PRESERVE, Irbesartan in Patients with Heart Failure and Preserved Ejection Fraction; MISCHF, Management to Improve Survival in Congestive Heart Failure; NHC, National Heart Care; OPTIMIZE-HF, Organized Program to Initiate Lifesaving Treatment in Hospitalized Patients with Heart Failure; OR, odds ratio; PEP-CHF, Perindopril in Elderly People with Chronic Heart Failure; PSM, propensity score matched study; RCT, randomized controlled trial; RR, risk ratio/relative risk; SOLVD, Studies of Left Ventricular Dysfunction; V-HeFT-II, Vasodilator Heart Failure Trial II; WAHMD, Western Australia Hospital Morbidity Data; X-SOLVD, Extended follow-up of the SOLVD trials.

**Table 3:** All-cause mortality in randomized and non-randomized beta-blocker HF studies

First author, country, year of publication (study name)	Study design	Study period	Region	Mean follow-up (months)	Patients (*n*)	Study (*n*)	Control (*n*)	All-cause mortality—unadjusted HR (95% CI)	All-cause mortality—adjusted HR (95% CI)
HFrEF									
Randomized controlled trials—beneficial treatment effect									
Packer, USA, 2001 (COPERNICUS)^128^	RCT	1997–2000	Multiregional	10	2289	1156	1133	RR: 0.65 (0.52–0.81; *P* < 0.00013)	—
MERIT-HF Study Group, Sweden, 1999 (MERIT-HF)^129^	RCT	1997–1998	Europe, USA	12	3991	1990	2001	RR: 0.66 (0.53–0.81; *P* < 0.0001)	—
CIBIS Investigators, UK, 1999 (CIBIS-II)^130^	RCT	—	Europe	16	2647	1327	1320	0.66 (0.54–0.81; *P* < 0.0001)	—
Packer, USA, 1996 (US Carvedilol HF Study Group)^131^	RCT	1993–1995	USA	7	1094	696	398	RR: 0.35 (0.20–0.61; *P* < 0.001)	—
Randomized controlled trials—neutral treatment effect									
van Veldhuisen, Netherlands, 2009 (SENIORS)^132^	Pre-specified subgroup analysis of RCT (EF <35%) (≥70 years)	2000–2002	Europe	21	1359	678	681	0.84 (0.66–1.08)	—
BEST Investigators, USA, 2001 (BEST)^133^	RCT	1995–1998	USA, Canada	24	2708	1354	1354	0.90 (0.78–1.02; *P* > 0.10)	—
ANZ HF Research Collaborative Group, New Zealand, 1997 (ANZ)^134^	RCT (IHD)	—	Australia, New Zealand	19	415	207	208	RR: 0.76 (0.42–1.36; *P* > 0.1)	—
CIBIS Investigators, France, 1994 (CIBIS-I)^135^	RCT	1989–1992	Europe	23	641	320	321	—	RR: 0.80 (0.56–1.15)
Observational studies—beneficial treatment effect									
Cadrin-Tourigny, Canada, 2017 (AF-CHF)^47^	*Post hoc* analysis of RCT (PSM) (AF)	2001–2005	Multiregional	37^a^	655	426	229	—	0.72 (0.55–0.95; *P* < 0.018)
Bhatia, USA, 2015 (Alabama HF Project)^48^	Retrospective cohort study (PSM) (≥65 years)	1998–2001	USA	48	760	380	380	—	0.81 (0.67–0.98)
Ushigome, Japan, 2015 (2. CHART-2)^30^	Prospective cohort study	2006–2010	Japan	36	1360	870	490	—	0.59 (0.44–0.81; *P* < 0.001)
Del Carlo, Brazil, 2014^49^	Retrospective cohort study	1992, 1994, 1996, 1999, 2005–2006	Brazil	12	333	199	134	0.3 (0.2–0.5; *P* < 0.001)	0.3 (0.2–0.5; *P* < 0.001)
Liu, China, 2014^28^	Prospective cohort study	2005–2010	China	52^a^	2154	1471	683	—	0.75 (0.57–0.999; *P* < 0.049)
Lund, Sweden, 2014 (Swedish HF Registry)^50^	Registry (PSM)	2005–2012	Sweden	23^a^	6081	4054	2027	—	0.89 (0.82–0.97; *P* < 0.005)
El-Refai, USA, 2013^51^	Retrospective cohort study	2000–2008	USA	25^a^	1094	927	167	—	0.26 (0.17–0.40; *P* < 0.001)
Xu, China, 2013^52^	Retrospective cohort study	2007–2012	China	31^a^	685	555	130	—	0.69 (0.50–0.95; *P* < 0.021)
Teng, Australia, 2010 (WAHMD)^46^	Retrospective cohort study	1996–2006	Australia	12	225	100	125	—	0.53 (0.32–0.87; *P* < 0.011)
Hernandez, USA, 2009 (OPTIMIZE-HF)^53^	Registry (≥65 years)	—	USA	12	3001	1800	1201	0.65 (0.57–0.73)	0.77 (0.68–0.87)
Miyagishima, Japan, 2009^54^	Retrospective cohort study	2000–2004	Japan	36	431	297	134	—	0.48 (0.32–0.73)
Fauchier, France, 2009 (41% HFrEF)^55^	Retrospective cohort study (AF)	2000–2004	France	29	1269	449	820	—	RR: 0.60 (0.40–0.89; *P* < 0.01)
Pascual-Figal, Spain, 2008^56^	Registry (>70 years)	2002–2003	Spain	31^a^	272	139	133	0.45 (0.31–0.65; *P* < 0.001)	0.53 (0.34–0.80; *P* < 0.003)
Jost, Germany, 2005 (Ludwigshafen HF Registry) (1. ‘Trial patients’)^57^	Registry	1995–2004	Germany	31	278	166	112	—	0.57 (0.38–0.86)
Jost, Germany, 2005 (Ludwigshafen HF Registry) (2. ‘Non-trial patients’)^57^	Registry	1995–2004	Germany	31	397	204	193	—	0.72 (0.53–0.97)
Bobbio, Italy, 2003 (BRING-UP)^58^	Prospective cohort study	1998	Italy	12	2843	1582	1261	RR: 0.46 (0.38–0.57)	0.64 (0.48–0.86)
Observational studies—neutral treatment effect									
Ushigome, Japan, 2015 (1. CHART-1)^30^	Prospective cohort study	2000–2005	Japan	36	543	184	359	—	0.87 (0.50–1.50; *P* < 0.610)
Huan Loh, UK, 2007^59^	Retrospective cohort study	—	UK	36^a^	900	738	162	0.54 (0.40–0.73; *P* < 0.001)	0.73 (0.53–1.02; *P* < 0.067)
HFpEF									
Randomized controlled trials—neutral treatment effect									
Yamamoto, Japan, 2013 (J-DHF)^136^	PROBE	2004–2009	Japan	38	245	120	125	0.99 (0.53–1.86; *P* < 0.975)	—
van Veldhuisen, Netherlands, 2009 (SENIORS)^132^	Pre-specified subgroup analysis of RCT (EF >35%) (≥70 years)	2000–2002	Europe	21	752	380	372	0.91 (0.62–1.33; *P* < 0.718)	—
Observational studies—beneficial treatment effect									
Ruiz, Spain, 2016^60^	Prospective cohort study (PSM)	2006–2015	Spain	22^a^	1970	985	985	RR: 0.76 (0.70–0.83; *P* < 0.001)	0.78 (0.71–0.85; *P* < 0.001)
Lund, Sweden, 2014 (Swedish HF Registry)^50^	Registry (PSM)	2005–2012	Sweden	23^a^	8244	5496	2748	—	0.93 (0.86–0.996; *P* < 0.04)
El-Refai, USA, 2013^51^	Retrospective cohort study	2000–2008	USA	25^a^	741	570	171	—	0.43 (0.27–0.68; *P* < 0.001)
Nevzorov, Israel, 2012^61^	Retrospective cohort study	2001–2005	Israel	24	345	154	191	—	0.69 (0.47–0.99; *P* < 0.046)
Gomez-Soto, Spain, 2011^62^	Prospective cohort study (propensity score adjusted)	2001–2005	Spain	30^a^	1085	378	707	RR: 0.37 (0.21–0.50; *P* < 0.001)	0.72 (0.58–0.84)
Teng, Australia, 2010 (WAHMD)^46^	Retrospective cohort study	1996–2006	Australia	12	284	101	183	—	0.62 (0.39–0.99; *P* < 0.048)
Fauchier, France, 2009 (35% HFpEF)^55^	Retrospective cohort study (AF)	2000–2004	France	29	1269	449	820	—	RR: 0.45 (0.26–0.80; *P* < 0.006)
Shah, USA, 2008 (NHC)^32^	Retrospective cohort study (≥65 years)	1998–1999, 2000–2001	USA	36	13 533	4562	8971	—	RR: 0.92 (0.87–0.97)
Dobre, Netherlands, 2007^63^	Prospective cohort study (propensity score adjusted)	2000–2005	Netherlands	25	443	227	216	—	0.57 (0.37–0.88; *P* < 0.01)
Observational studies—neutral treatment effect									
Ushigome, Japan, 2015 (1. CHART-1)^30^	Prospective cohort study	2000–2005	Japan	36	463	104	359	—	0.89 (0.45–1.75; *P* < 0.734)
Ushigome, Japan, 2015 (2. CHART-2)^30^	Prospective cohort study	2006–2010	Japan	36	2316	1018	1298	—	0.94 (0.73–1.22; *P* < 0.654)
Patel, USA, 2014 (OPTIMIZE-HF)^64^	Registry (PSM) (≥65 years)	2003–2004	USA	72	2198	1099	1099	—	0.99 (0.90–1.10; *P* < 0.897)
Hernandez, USA, 2009 (OPTIMIZE-HF)^53^	Registry (≥65 years)	—	USA	12	4153	1621	2532	0.87 (0.77–0.97)	0.94 (0.84–1.07)
Mixed/unspecified HF phenotype									
Randomized controlled trials—neutral effect									
Flather, UK, 2005 (SENIORS) (65% HFrEF, 35% HFpEF)^137^	RCT (≥70 years)	2000–2002	Multiregional	21	2128	1067	1061	0.88 (0.71–1.08; *P* < 0.21)	—
Observational studies—beneficial treatment effect									
Katz, Israel, 2016 (HFSIS) (38% HFrEF, 15% HFmrEF, 22% HFpEF, 26% unknown)^65^	Prospective cohort study	2003	Israel	120	2402	1481	921	—	0.83 (0.77–0.89; *P* < 0.001)
Maison, France, 2013^66^	Registry (propensity score adjusted)	2000	France	96	281	101	180	—	0.54 (0.34–0.84)
Gastelurrutia, Spain, 2012 (75% HFrEF, 25% HFrEF)^45^	Prospective cohort study	2001–2008	Spain	44^a^	960	776	184	—	0.51 (0.39–0.66; *P* < 0.001)
Marijon, France, 2010 (EVADEF)^67^	Prospective cohort study (ICD)	2001–2003	France	22	1030	721	309	0.53 (0.30–0.91; *P* < 0.02)	0.56 (0.32–0.98; *P* < 0.04)
Teng, Australia, 2010 (WAHMD) (24% HFrEF, 30% HFpEF, 46% unknown)^46^	Retrospective cohort study	1996–2006	Australia	12	944	318	626	—	0.68 (0.53–0.86; *P* < 0.002)
Fauchier, France, 2009 (41% HFrEF, 35% HFpEF, 24% unknown)^55^	Retrospective cohort study (AF)	2000–2004	France	29	1269	449	820	0.59 (0.45–0.78; *P* < 0.0002)	0.60 (0.43–0.84; *P* < 0.003)
Jordán, Spain, 2009 (BADAPIC) (77% HFrEF, 23% HFpEF)^68^	Registry	2000–2002	Spain	35	3162	2242	920	—	RR: 0.82 (0.47–0.95)
Dobre, Netherlands, 2007 (55% HFrEF, 45% HFpEF)^69^	Prospective cohort study (propensity score adjusted)	2000–2004	Netherlands	22	625	308	317		0.55 (0.39–0.78; *P* < 0.001)
Keyhan, Canada, 2007 (1. female cohort)^70^	Retrospective cohort study (≥65 years)	1998–2003	Canada	30	14 693	7584	7109	0.67 (0.64–0.70)	0.79 (0.75–0.83)
Keyhan, Canada, 2007 (2. male cohort)^70^	Retrospective cohort study (≥65 years)	1998–2003	Canada	30	13 144	6499	6645	0.64 (0.61–0.67)	0.76 (0.72–0.80)
Chan, USA, 2005 (CHS) (19% HFrEF, 36% HFpEF, 45% unknown)^71^	Prospective cohort study (≥65 years)	1989–2000	USA	120	950	157	793	0.74 (0.56–0.98)	0.74 (0.56–0.98)
Tandon, Canada, 2004 (75% HFrEF, 25% HFpEF)^41^	Prospective cohort study	1989–2001	Canada	32^a^	1041	475	566	—	OR: 0.52 (0.39–0.70)
Maggioni, Italy, 2003 (BRING-UP) (1. no BB vs. continued BB)^72^	Registry	1998	Italy	12	2226	771	1455	—	0.74 (0.55–0.99; *P* < 0.045)
Maggioni, Italy, 2003 (BRING-UP) (2. no BB vs. initiated BB)^72^	Registry	1998	Italy	12	2320	865	1455	—	0.60 (0.45–0.80; *P* < 0.0003)
McCullough, USA, 2003 (REACH)^73^	Retrospective cohort study	1995–1998	USA	12	1317	647	670	—	OR: 0.75 (0.57–0.98; *P* < 0.04)
Sin, Canada, 2002 (19% HFrEF, 36% HFpEF, 45% unknown)^44^	Retrospective cohort study (≥65 years) (propensity score adjusted)	1994–1998	Canada	21^a^	11 942	1162	10 780	—	0.72 (0.65–0.80)
McAlister, Canada, 1999 (78% HFrEF, 22% HFpEF)^74^	Prospective cohort study	1989–1995	Canada	17	566	147	419	—	OR: 0.5 (*P* < 0.006–95% CI not reported)
Observational studies—neutral treatment effect									
Ushigome, Japan, 2015 (1. CHART-1) (54% HFrEF, 46% HFpEF)^30^	Prospective cohort study	2000–2005	Japan	36	1006	288	718	—	0.96 (0.63–1.44; *P* < 0.829)
Ushigome, Japan, 2015 (1. CHART-2) (37% HFrEF, 63% HFpEF)^30^	Prospective cohort study	2006–2010	Japan	36	3676	1886	1790	—	0.82 (0.68–1.00; *P* < 0.055)

a Median.

—, Not reported; AF, atrial fibrillation cohort; AF-CHF, Atrial Fibrillation and Congestive Heart Failure; ANZ, Australia/New Zealand; BADAPIC, Registry of the Working Group on Heart Failure, Heart Transplantation and Other Therapeutic Alternatives of the Spanish Society of Cardiology; BB, beta-blocker; BEST, Beta-blocker Evaluation in Survival Trial; BRING-UP: Beta-Blockers in Patients With Congestive Heart Failure: Guided Use in Clinical Practice; CHS, Cardiovascular Health Study; CHART, Chronic Heart Failure Analysis and Registry in the Tohoku district; CI, confidence interval; CIBIS, Cardiac Insufficiency Bisoprolol Study; COPERNICUS, Carvedilol Prospective Randomized Cumulative Survival; EF, ejection fraction; EVADEF: Évaluation Médico-Économique du Défibrillateur Automatique Implantable; HF, heart failure; HFmrEF, heart failure with mid-range ejection fraction; HFpEF, heart failure with preserved ejection fraction; HFrEF, heart failure with reduced ejection fraction; HFSIS, National Heart Failure Survey in Israel; HR, hazard ratio; ICD, implantable cardioverter defibrillator cohort; IHD, ischaemic heart disease cohort; J-DHF, Japanese Diastolic Heart Failure; MERIT-HF, Metoprolol CR/XL Randomised Intervention Trial in Congestive Heart Failure; NHC, National Heart Care; OPTIMIZE-HF, Organized Program to Initiate Lifesaving Treatment in Hospitalized Patients with Heart Failure; OR, odds ratio; PROBE, prospective randomized open blind endpoint study; PSM, propensity score matched study; RCT, randomized controlled trial; REACH, Resource Utilization Among Congestive Heart Failure; RR, risk ratio/relative risk; SENIORS, Study of the Effects of Nebivolol Intervention on Outcomes and Rehospitalisation in Seniors with Heart Failure; ‘Trial patients’, patients meeting the inclusion criteria of the MERIT-HF trial; ‘'Non-trial patients’, patients not meeting the inclusion criteria of the MERIT-HF trial; WAHMD, Western Australia Hospital Morbidity Data.

**Table 4:** All-cause mortality in randomized and non-randomized MRA HF studies

First author, country, year of publication (study name)	Study design	Study period	Region	Mean follow-up (months)	Patients (*n*)	Study (*n*)	Control (*n*)	All-cause mortality—unadjusted HR (95% CI)	All-cause mortality—adjusted HR (95% CI)
HFrEF									
Randomized controlled trials—beneficial treatment effect									
Zannad, USA, 2011 (EMPHASIS-HF)^138^	RCT	2006–2010	Multiregional	21^a^	2737	1364	1373	0.78 (0.64–0.95; *P* < 0.01)	0.76 (0.62–0.93; *P* < 0.008)
Pitt, USA, 1999 (RALES)^139^	RCT	1995–1996	Multiregional	24	1663	822	841	RR: 0.70 (0.60–0.82; *P* < 0.001)	—
Observational studies—beneficial treatment effect									
Hamaguchi, Japan, 2010 (JCARE-CARD)^75^	Prospective cohort study	2004–2005	Japan	26	946	435	511	0.75 (0.54–1.04; *P* < 0.078)	0.62 (0.41–0.93; *P* < 0.02)
Observational studies—neutral treatment effect									
Lam, USA, 2017 (Alabama HF Project)^76^	Retrospective cohort study (PSM)	1998–2001	USA	12	648	324	324	—	1.11 (0.83–1.49; *P* < 0.483)
Ushigome, Japan, 2015 (1. CHART-1)^30^	Prospective cohort study	2000–2005	Japan	36	543	116	427	—	1.39 (0.80–2.43; *P* < 0.247)
Ushigome, Japan, 2015 (2. CHART-2)^30^	Prospective cohort study	2006–2010	Japan	36	1360	493	867	—	1.23 (0.91–1.66; *P* < 0.172)
Frankenstein, Norway, 2013 (Norwegian HF Registry)^77^	Registry (PSM)	—	Norway, Germany	44	4832	1565	3267	1.08 (0.97–1.22; *P* < 0.17)	1.03 (0.88–1.20; * P* < 0.74)
Lee, USA, 2013 (KPNC)^78^	Retrospective cohort study	2006–2008	USA	30^a^	2358	521	1837	—	0.93 (0.60–1.44)
Lund, Sweden, 2013 (Swedish HF Registry)^79^	Registry (PSM)	2000–2012	Sweden	27^a^	18 852	6551	12 301	1.10 (1.04–1.15; *P* < 0.001)	1.05 (1.00–1.11; *P* < 0.054)
Pascual-Figal, Spain, 2013 (MUSIC)^80^	Prospective cohort study (PSM)	2003–2004	Spain	38^a^	362	181	181	1.25 (0.81–1.94; *P* < 0.318)	1.46 (0.84–2.55; *P* < 0.185)
Hernandez, USA, 2012 (GWTG-HF/Medicare)^81^	Registry	2005–2009	USA	36	5887	1070	4817	0.98 (0.90–1.06; * P* < 0.58)	1.05 (0.97–1.15; *P* < 0.23)
Miyagishima, Japan, 2009^54^	Retrospective cohort study	2000–2004	Japan	36	431	312	119	—	0.83 (0.54–1.30)
Ouzounian, Canada, 2007 (ICONS)^82^	Prospective cohort study	1997–2001	Canada	24	7816	644	7172	—	OR: 0.97 (0.79–1.20)
Observational studies—harmful treatment effect									
O'Meara, Canada, 2012 (AF-CHF)^83^	*Post hoc* analysis of RCT (AF)	2001–2005	Multiregional	37	1376	616	760	—	1.40 (1.10–1.80; *P* < 0.005)
HFpEF									
Randomized controlled trials—neutral treatment effect									
Pfeffer, USA, 2015 (TOPCAT-Americas subgroup)^140^	*Post hoc* analysis of RCT	2006–2012	USA, Canada, Brazil, Argentina	35	1767	886	881	0.83 (0.68–1.02; *P* < 0.08)	—
Pfeffer, USA, 2015 (TOPCAT-Russia/Georgia subgroup)^140^	*Post hoc* analysis of RCT	2006–2012	Russia, Georgia	44	1678	836	842	1.12 (0.80–1.55; *P* < 0.51	—
Pitt, USA, 2014 (TOPCAT)^141^	RCT	2006–2012	Multiregional	40	3445	1722	1723	0.91 (0.77–1.08; *P* < 0.295)	0.88 (0.74–1.05; *P* < 0.151)
Observational studies—beneficial treatment effect									
Bonsu, Malaysia, 2017^84^	Retrospective cohort study	2009–2013	Ghana	60	878	227	651	—	0.66 (0.49–0.89; *P* < 0.006)
Observational studies—neutral treatment effect									
Ushigome, Japan, 2015 (2. CHART-2)^30^	Prospective cohort study	2006–2010	Japan	36	2316	491	1825	—	0.96 (0.72–1.29; *P* < 0.808)
Patel, USA, 2013 (OPTIMIZE-HF)^85^	Registry (PSM) (≥65 years)	2002–2008	USA	29	974	487	487	—	1.03 (0.89–1.20; *P* < 0.693)
Mixed/unspecified HF phenotype									
Observational studies—beneficial treatment effect									
Bonsu, Malaysia, 2017 (23% HFrEF, 18% HFmrEF, 59% HFpEF)^84^	Retrospective cohort study	2009–2013	Ghana	60	1488	417	1071	—	0.81 (0.65–0.99; *P* < 0.049)
Sligl, Canada, 2004 (75% HFrEF, 25% HFpEF)^86^	Prospective cohort study	1989–2001	Canada	32^a^	1037	136	901	—	RR: 0.13 (0.04–0.42)
Observational studies—neutral treatment effect									
Ushigome, Japan, 2015 (1. CHART-1) (54% HFrEF, 46% HFpEF)^30^	Prospective cohort study	2000–2005	Japan	36	1006	182	824	—	1.36 (0.89–2.07; *P* < 0.154)
Ushigome, Japan, 2015 (2. CHART-2) (37% HFrEF, 63% HFpEF)^30^	Prospective cohort study	2006–2010	Japan	36	3676	984	2692	—	1.14 (0.93–1.39; *P* < 0.223)
Teng, Australia, 2010 (34% HFrEF, 19% HFpEF, 47% unknown)^46^	Retrospective cohort study	1996–2006	Australia	12	944	154	790	—	0.87 (0.64–1.20; *P* < 0.390)

a Median.

—, Not reported; AF, atrial fibrillation cohort; AF-CHF, Atrial Fibrillation and Congestive Heart Failure; CHART, Chronic Heart Failure Analysis and Registry in the Tohoku district; CI, confidence interval; EMPHASIS-HF, Eplerenone in Mild Patients Hospitalization and Survival Study in Heart Failure; GWTG-HF, Get With The Guidelines Heart Failure; HF, heart failure; HFmrEF, heart failure with mid-range ejection fraction; HFpEF, heart failure with preserved ejection fraction; HFrEF, heart failure with reduced ejection fraction; HR, hazard ratio; ICONS, Improving Cardiovascular Outcomes in Nova Scotia; JCARE-CARD, Japanese Cardiac Registry of Heart Failure in Cardiology; KPNC, Kaiser Permanente Northern California; MRA, mineralocorticoid receptor antagonist; MUSIC, Multi-Sensor Monitoring in Congestive Heart Failure; OPTIMIZE-HF, Organized Program to Initiate Lifesaving Treatment in Hospitalized Patients with Heart Failure; OR, odds ratio; PSM, propensity score matched study; RALES, Randomized Aldactone Evaluation Study; RCT, randomized controlled trial; RR, risk ratio/relative risk; TOPCAT, Treatment of Preserved Cardiac Function Heart Failure with an Aldosterone Antagonist Trial.

**Table 5:** All-cause mortality in randomized and non-randomized statin HF studies

First author, country, year of publication (study name)	Study design	Study period	Region	Mean follow-up (months)	Patients (*n*)	Study (*n*)	Control (*n*)	All-cause mortality—unadjusted HR (95% CI)	All-cause mortality—adjusted HR (95% CI)
HFrEF									
Randomized controlled trials—neutral treatment effect									
Kjekshus, Norway, 2007 (CORONA)^142^	RCT	2003–2005	Europe, Russia, South Africa	33^a^	5011	2514	2497	0.95 (0.86–1.05; *P* < 0.31)	—
Takano, Japan, 2013 (PEARL)^143^	PROBE	2006–2008	Japan	36^a^	574	288	286	—	0.73 (0.44–1.20; *P* < 0.211)
Observational studies—beneficial treatment effect									
Alehagen, Sweden, 2015 (Swedish HF Registry)^87^	Registry (PSM)	2000–2012	Sweden	47^a^	10 762	5381	5381	—	0.81 (0.76–0.86; *P* < 0.001)
Liu, China, 2014^28^	Prospective cohort study	2005–2010	China	52^a^	2154	936	1218	—	0.50 (0.37–0.67; *P* < 0.001)
Gomez-Soto, Spain, 2010 (56% HFrEF)^88^	Prospective cohort study (propensity score adjusted)	2001–2005	Spain	34	2573	1343	1230	—	0.20 (0.09–0.31; *P* < 0.001)
Sumner, USA, 2009 (COMPANION)^89^	*Post hoc* analysis of RCT (CRT)	2000–2002	USA	15–16^a^	1520	603	917	0.85 (0.67–1.07; *P* < 0.15)	0.77 (0.61–0.97; *P* < 0.03)
Coleman, USA, 2008^90^	Retrospective cohort study (ICD)	1997–2007	USA	31	1204	642	562	—	0.67 (0.53–0.85; *P* < 0.001)
Dickinson, USA, 2007 (SCD-HeFT)^91^	*Post hoc* analysis of RCT	1997–2001	North America, New Zealand	46	2521	965	1556	—	0.70 (0.58–0.83; *P* < 0.001)
Huan Loh, UK, 2007 (1. no statin vs. initiated statin)^59^	Retrospective cohort study	—	UK	36^a^	479	102	377	0.52 (0.32–0.84)	0.50 (0.30–0.83)
Krum, Australia, 2007 (CIBIS-II)^92^	*Post hoc* analysis of RCT	—	Europe	16	2647	226	2421	0.57 (0.37–0.94)	0.60 (0.39–0.94); *P* < 0.02
Krum, Australia, 2007 (Val-HeFT)^93^	*Post hoc* analysis of RCT	1997–1999	Multiregional	23	5010	1602	3408	—	0.81 (0.70–0.94; *P* < 0.005)
Anker, UK, 2006 (1. ELITE-II)^94^	*Post hoc* analysis of RCT	1997–1998	Multiregional	18^a^	3132	2734	398	0.61 (0.45–0.83; *P* < 0.0007)	0.61 (0.44–0.84; *P* < 0.003)
Anker, UK, 2006 (2. European Centres Study)^94^	Retrospective cohort study	1992–2000	Europe	24^a^	2068	705	1363	0.59 (0.49–0.72; *P* < 0.0001)	0.58 (0.44–0.77; *P* < 0.0001)
Goldberger, USA, 2006 (DEFINITE)^95^	*Post hoc* analysis of RCT (non-ischaemic DCM)	1998–2002	USA	29	458	110	348	0.22 (0.09–0.55; *P* < 0.001)	0.23 (0.09–0.58; *P* < 0.04)
Ray, Canada, 2005^96^	Retrospective cohort study (66–85 years)	1995–2001	Canada	24	28 828	1146	27 682	0.50 (0.43–0.59)	0.67 (0.57–0.78)
Mozaffarian, USA, 2004 (PRAISE)^97^	*Post hoc* analysis of RCT	1992–1994	USA	15	1153	134	1019	0.38 (0.23–0.64)	0.44 (0.26–0.75)
Observational studies—neutral treatment effect									
Ushigome, Japan, 2015 (CHART-2)^30^	Prospective cohort study	2006–2010	Japan	36	1360	515	845	—	0.84 (0.60–1.17; *P* < 0.299)
Ouzounian, Canada, 2009 (EFFECT) (23% HFrEF)^98^	Retrospective cohort study	1999–2001	Canada	60	6451	5330	1121	—	0.84 (0.70–1.02; *P* < 0.07)
Huan Loh, UK, 2007 (2. no statin vs. continued statin)^59^	Retrospective cohort study	—	UK	36^a^	760	377	383	0.74 (0.52–1.05)	0.82 (0.55–1.23)
Mixed/unspecified HF phenotype									
Randomized controlled trials—neutral treatment effect									
Tavazzi, Italy, 2008 (GISSI-HF Rosuvastatin) (90% HFrEF, 10% HFpEF)^144^	RCT (≥60 years)	2002–2005	Italy	47^a^	4574	2285	2289	1.03 (95.5% CI 0.92–1.15; *P* < 0.660)	1.00 (95.5% CI 0.90–1.12; *P* < 0.943)
Observational studies—beneficial treatment effect									
Bonsu, Malaysia, 2017 (23% HFrEF, 18% HFmrEF, 59% HFpEF)^99^	Retrospective cohort study (IPTW)	2009–2013	Ghana	60	1488	552	936	—	0.79 (0.65–0.96; *P* < 0.019)
Ballo, Italy, 2016^100^	Retrospective cohort study	—	Italy	12	2088	643	1445	—	0.65 (0.51–0.83; *P* < 0.001)
Gastelurrutia, Spain, 2012 (75% HFrEF, 25% HFrEF)^45^	Prospective cohort study	2001–2008	Spain	44^a^	960	591	369	0.45 (0.37–0.54; *P* < 0.001)	0.66 (0.53–0.83; *P* < 0.001)
Gomez-Soto, Spain, 2010 (56% HFrEF, 44% HFpEF)^88^	Prospective cohort study (propensity score adjusted)	2001–2005	Spain	34	2573	1343	1230	—	0.71 (0.59–0.83)
Jordán, Spain, 2009 (BADAPIC) (77% HFrEF, 23% HFpEF)^68^	Registry	2000–2002	Spain	35	3162	1305	1857	—	RR: 0.73 (0.45–0.88; *P* < 0.001)
Nevzorov, Israel, 2009 (61% HFrEF, 39% HFpEF)^101^	Retrospective cohort study (IHD)	2001–2005	Israel	12	656	238	418	OR: 0.63 (0.40–0.87; *P* < 0.006)	0.66 (0.40–0.97; *P* < 0.035)
Ouzounian, Canada, 2009 (EFFECT)^98^	Retrospective cohort study (PSM)	1999–2001	Canada	60	1442	721	721	—	0.85 (0.72–1.00; *P* < 0.05)
Ryan, UK, 2009 (THIN) (1. statin before HF diagnosis)^102^	Retrospective cohort study	1995–2004	UK	24	10 914	2185	8239	—	0.53 (0.40–0.70; *P* < 0.001)
Ryan, UK, 2009 (THIN) (2. statin after HF diagnosis)^102^	Retrospective cohort study	1995–2004	UK	24	8729	191	8538	—	0.68 (0.46–0.99; *P* < 0.047)
Foody, USA, 2006 (NHC) (48% HFrEF, 52% HFpEF)^103^	Retrospective cohort study (≥65 years)	1998–1999, 2000–2001	USA	36^a^	54 960	9163	45 797	0.67 (0.65–0.69; *P* < 0.001)	0.82 (0.79–0.85; *P* < 0.001)
Go, USA, 2006 (KPNC) (25% HFrEF, 26% HFpEF, 49% unknown)^104^	Retrospective cohort study (propensity score adjusted)	1996–2004	USA	29^a^	24 598	12 648	11 960	—	0.76 (0.72–0.80; *P* < 0.001)
Observational studies—neutral treatment effect									
Ushigome, Japan, 2015 (CHART-2) (37% HFrEF, 63% HFpEF)^30^	Prospective cohort study	2006–2010	Japan	36	3676	1332	2344	—	0.81 (0.65–1.02; *P* < 0.068)

a Median.

—, Not reported; BADAPIC, Registry of the Working Group on Heart Failure, Heart Transplantation and Other Therapeutic Alternatives of the Spanish Society of Cardiology; CHART, Chronic Heart Failure Analysis and Registry in the Tohoku district; CI, confidence interval; CIBIS-II, Cardiac Insufficiency Bisoprolol Study II; COMPANION, Comparison of Medical Therapy, Pacing, and Defibrillation in Heart Failure; CORONA, Controlled Rosuvastatin Multinational Trial in Heart Failure; CRT, cardiac resynchronization therapy cohort; DCM, dilated cardiomyopathy cohort; DEFINITE, Defibrillators in Non-Ischaemic Cardiomyopathy Treatment Evaluation; EFFECT, Enhanced Feedback for Effective Cardiac Treatment; ELITE-II, Evaluation of Losartan in the Elderly II; GISSI-HF, Gruppo Italiano per lo Studio della Sopravvivenza nell'Insuffi cienza cardiaca Heart Failure; HF, heart failure; HFmrEF, heart failure with mid-range ejection fraction; HFpEF, heart failure with preserved ejection fraction; HFrEF, heart failure with reduced ejection fraction; HR, hazard ratio; ICD, implantable cardioverter defibrillator cohort; IHD, ischaemic heart disease cohort; IPTW, inverse-probability-of-treatment weighted study; KPNC, Kaiser Permanente Northern California; NHC, National Heart Care; OR, odds ratio; PEARL, Pitavastatin Heart Failure study; PRAISE, Prospective Randomized Amlodipine Survival Evaluation; PROBE, prospective randomized open blind endpoint study; PSM, propensity score matched study; RCT, randomized controlled trial; RR, risk ratio/relative risk; SCD-HeFT, Sudden Cardiac Death in Heart Failure Trial; THIN, The Health Improvement Network; Val-HeFT, Valsartan Heart Failure Trial.

**Table 6:** All-cause mortality in randomized and non-randomized digoxin HF studies

First author, country, year (study name)	Study design	Study period	Region	Mean follow-up (months)	Patients (*n*)	Study (*n*)	Control (*n*)	All-cause mortality—unadjusted HR (95% CI)	All-cause mortality—adjusted HR (95% CI)
HFrEF									
Randomized controlled trials—neutral treatment effect									
Digoxin Investigation Group, USA, 1997 (DIG Main Trial)^145^	RCT (SR)	1991–1993	USA, Canada	37	6800	3397	3403	RR: 0.99 (0.91–1.07; *P* < 0.80)	—
Observational studies—beneficial treatment effect									
Andrey, Spain, 2011 (51% HFrEF)^105^	Prospective cohort study (PSM) (SR/AF)	2001–2008	Spain	46^a^	2842	1421	1421	—	0.92 (0.89–0.95; *P* < 0.005)
Observational studies—neutral treatment effect									
Ushigome, Japan, 2015 (1. CHART-1)^30^	Prospective cohort study (SR/AF)	2000–2005	Japan	36	543	229	314	—	0.99 (0.61–1.61; *P* < 0.978)
Ushigome, Japan, 2015 (2. CHART-2)^30^	Prospective cohort study (SR/AF)	2006–2010	Japan	36	1360	586	774	—	1.10 (0.80–1.51; *P* < 0.558)
Fauchier, France, 2009 (41% HFrEF)^55^	Retrospective cohort study (AF)	2000–2004	France	29	1269	591	678	—	RR: 0.79 (0.54–1.16; *P* < 0.23)
Dhaliwal, USA, 2008^106^	Retrospective cohort study (SR/AF)	2002–2004	USA	10^a^	347	155	192	1.15 (0.85–1.55; *P* < 0.371)	1.11 (0.81–1.53; *P* < 0.521)
Observational studies—harmful treatment effect									
Al-Khateeb, Saudi Arabia, 2017^107^	Retrospective cohort study (PSM) (SR/AF)	2000–2015	Saudi Arabia	43^a^	1075	325	750	1.81 (1.33–2.45; *P* < 0.001)	1.74 (1.20–2.38; *P* < 0.0001)
Freeman, USA, 2013 (KPNC)^108^	Retrospective cohort study (SR/AF)	2006–2008	USA	30^a^	2891	529	2362	—	1.72 (1.25–2.36)
Butler, USA, 2010 (Val-HeFT)^109^	*Post hoc* analysis of RCT (SR/AF)	—	Multiregional	23	5010	1636	3374	1.46 (1.23–1.64; *P* < 0.001)	1.28 (1.05–1.57; *P* < 0.02)
Domanski, USA, 2005 (SOLVD) (1. female cohort)^110^	*Post hoc* analysis of RCT (SR/AF)	1986–1989	USA, Canada, Belgium	39	988	370	618	1.48 (1.10–2.00; *P* < 0.01)	1.36 (1.03–1.80; *P* < 0.03)
Domanski, USA, 2005 (SOLVD) (2. male cohort)^110^	*Post hoc* analysis of RCT (SR/AF)	1986–1989	USA, Canada, Belgium	39	5809	1874	3935	1.37 (1.20–1.56; *P* < 0.0001)	1.42 (1.26–1.61; *P* < 0.0001)
HFpEF									
Randomized controlled trials—neutral treatment effect									
Ahmed, USA, 2006 (DIG Ancillary Trial)^146^	RCT (SR)	1991–1993	USA, Canada	37	988	492	496	0.99 (0.76–1.28; *P* < 0.925)	—
Observational studies—beneficial treatment effect									
Andrey, Spain, 2011 (49% HFpEF)^105^	Prospective cohort study (PSM) (SR/AF)	2001–2008	Spain	46^a^	2842	1421	1421	—	0.86 (0.79–0.92; *P* < 0.008)
Observational studies—neutral treatment effect									
Ushigome, Japan, 2015 (1. CHART-1)^30^	Prospective cohort study (SR/AF)	2000–2005	Japan	36	463	249	214	—	0.92 (0.55–1.54; *P* < 0.764)
Ushigome, Japan, 2015 (2. CHART-2)^30^	Prospective cohort study (SR/AF)	2006–2010	Japan	36	2316	335	1981	—	1.07 (0.81–1.41; *P* < 0.632)
Fauchier, France, 2009 (35% HFpEF)^55^	Retrospective cohort study (AF)	2000–2004	France	29	1269	591	678	—	RR: 1.21 (0.77–1.89; *P* < 0.42)
Mixed/unspecified HF phenotype									
Randomized controlled trials—neutral treatment effect									
Rich, USA, 2001 (DIG Overall)^147^	RCT (SR)	1991–1993	USA, Canada	37	7788	3889	3899	RR: 0.99 (0.92–1.07; *P* < 0.7815)	—
Observational studies—beneficial treatment effect									
Ahmed, USA, 2014 (Alabama HF Project) (57% HFrEF, 25% HFpEF, 18% unknown)^111^	Retrospective cohort study (PSM) (SR/AF)	1998–2001	USA	12	1842	921	921	—	0.83 (0.70–0.98)
Andrey, Spain, 2011 (51% HFrEF, 49% HFpEF)^105^	Prospective cohort study (PSM) (SR/AF)	2001–2008	Spain	46^a^	2842	1421	1421	—	0.90 (0.84–0.97)
Observational studies—neutral treatment effect									
Ushigome, Japan, 2015 (1. CHART-1) (54% HFrEF, 46% HFpEF)^30^	Prospective cohort study (SR/AF)	2000–2005	Japan	36	1006	478	528	—	0.97 (0.69–1.38; *P* < 0.875)
Ushigome, Japan, 2015 (2. CHART-2) (37% HFrEF, 63% HFpEF)^30^	Prospective cohort study (SR/AF)	2006–2010	Japan	36	3676	921	2755	—	1.06 (0.87–1.31; *P* < 0.555)
Flory, USA, 2012 (THIN) (1. female cohort)^112^	Retrospective cohort study (SR/AF)	1986–2008	UK	—	30 035	10 808	19 227	—	1.00 (0.96–1.06)
Flory, USA, 2012 (THIN) (2. male cohort)^112^	Retrospective cohort study (SR/AF)	1986–2008	UK	—	27 194	9487	17 707	—	1.00 (0.95–1.06)
Fauchier, France, 2009 (41% HFrEF, 35% HFpEF, 24% unknown)^55^	Retrospective cohort study (AF)	2000–2004	France	29	1269	591	678	—	0.90 (0.66–1.24; *P* < 0.53)
Hallberg, Sweden, 2007 (RIKS-HIA) (58% HFrEF, 42% HFpEF) (1. AF cohort)^113^	Registry (propensity score adjusted)	1995–2003	Sweden	12	16 960	7758	9202	RR: 1.07 (1.01–1.14)	RR: 1.00 (0.94–1.06)
Pedone, Italy, 2004 (GIFA)^42^	Prospective cohort study (SR/AF)	1998	Italy	10	818	539	279	—	0.75 (0.51–1.10)
Observational studies—harmful treatment effect									
Eisen, USA, 2017 (ENGAGE AF-TIMI 48) (41% HFrEF, 34% HFpEF, 24% unknown)^114^	*Post hoc* analysis of RCT (IPTW) (AF)	2008–2010	Multiregional	34^a^	8102	4051	4051	—	1.29 (1.15–1.44)
Katz, Israel, 2016 (HFSIS) (38% HFrEF, 15% HFmrEF, 22% HFpEF, 26% unknown)^65^	Prospective cohort study (SR/AF)	2003	Israel	120	2402	380	2022	—	1.27 (1.16–1.42; *P* < 0.001)
Madelaire, Denmark, 2016^115^	Retrospective cohort study (PSM) (SR)	1996–2012	Denmark	32^a^	15 981	5327	10 654	—	1.19 (1.15–1.24; *P* < 0.001)
Shah, Canada, 2014^116^	Retrospective cohort study (PSM) (≥65 years) (AF)	1998–2012	Canada	37	27 972	13 986	13 986	1.14 (1.11–1.17)	1.14 (1.10–1.17)
Whitbeck, USA, 2013 (AFFIRM)^117^	*Post hoc* analysis of RCT (AF)	—	Multiregional	42	1076	—	—	—	1.41 (1.09–1.84; *P* < 0.01)
Hallberg, Sweden, 2007 (RIKS-HIA) (58% HFrEF, 42% HFpEF) (2. SR cohort)^113^	Registry (propensity score adjusted)	1995–2003	Sweden	12	22 345	3796	18 549	RR: 1.35 (1.26–1.44)	RR: 1.11 (1.04–1.19)
Tandon, Canada, 2004 (75% HFrEF, 25% HFpEF)^41^	Prospective cohort study (SR/AF)	1989–2001	Canada	32^a^	1041	671	370	—	OR: 1.51 (1.10–2.07)

a Median.

—, Not reported; AF, atrial fibrillation cohort; AFFIRM, Atrial Fibrillation Follow-up Investigation of Rhythm Management; CHART, Chronic Heart Failure Analysis and Registry in the Tohoku district; CI, confidence interval; DIG, Digitalis Investigation Group; ENGAGE AF-TIMI 48, Effective Anticoagulation with Factor Xa Next Generation in Atrial Fibrillation - Thrombolysis in Myocardial Infarction 48; GIFA, Gruppo Italiano di Farmacovigilanza nell'Anziano; HF, heart failure; HFmrEF, heart failure with mid-range ejection fraction; HFpEF, heart failure with preserved ejection fraction; HFrEF, heart failure with reduced ejection fraction; HFSIS, National Heart Failure Survey in Israel; HR, hazard ratio; KPNC, Kaiser Permanente Northern California; IPTW, inverse-probability-of-treatment weighted study; OR, odds ratio; PSM, propensity score matched study; RCT, randomized controlled trial; RIKS-HIA, Registry of Information and Knowledge about Swedish Heart Intensive Care Admissions; RR, risk ratio/relative risk; SOLVD, Studies of Left Ventricular Dysfunction; SR, sinus rhythm cohort; SR/AF, sinus rhythm and atrial fibrillation cohort; THIN, The Health Improvement Network; Val-HeFT, Valsartan Heart Failure Trial.

### Angiotensin-converting enzyme inhibitors and angiotensin receptor blockers

#### Heart failure with reduced ejection fraction

Two landmark randomized trials in heart failure with reduced ejection fraction (HFrEF) demonstrated a reduction in mortality with an ACEI^118–120^ and one further trial showed a consistent benefit with an ARB.^121^ We identified one non-randomized study showing lower mortality in patients with HFrEF treated with an ACEI.^26^ Most studies, however, examined patients treated with either an ACEI or ARB. Of six such studies, four reported an association between ACEI/ARB use and lower mortality,^26–29^ whereas two did not.^30^ Overall, therefore, in HFrEF five non-randomized estimates of treatment ‘effect’ found that use of an ACEI or ARB was associated with lower mortality and two did not (*Table 2*).

#### Heart failure with preserved ejection fraction

One moderately large randomized trial showed no effect of perindopril on mortality, although the estimate of treatment effect was not robust because of limited power.^122^ However, two large RCTs showed no effect of irbesartan^123^ and candesartan (in Candesartan in Heart failure: Assessment of Reduction in Mortality and morbidity—CHARM)^124^ on mortality. Of eight observational studies examining ACEI use and outcome in heart failure with preserved ejection fraction (HFpEF), four suggested that use of this treatment was associated with a lower mortality,^31–34^ whilst four did not not^35–38^ (*Table 2*). We identified one observational study of ARB use in patients with HFpEF which suggested no mortality benefit.^39^ A further three non-randomized studies reported estimates of a treatment ‘effect’ for use of either an ACEI or ARB in HFpEF. One study found an association between ACEI/ARB use and better survival^29^ and two studies did not.^30^ Overall, therefore, in HFpEF, five non-randomized studies found that use of an ACEI or ARB was associated with lower mortality and seven did not (*Table 2*).

#### Mixed/unspecified heart failure phenotype

The CHARM Programme showed a neutral effect of candesartan on mortality in patients with HFpEF and HFrEF combined.^127^ Nine non-randomized studies were identified, which reported 10 estimates of a ‘treatment-effect’ for use of either an ACEI or ARB in patients with HFrEF or HFpEF (i.e. both major HF phenotypes). Of these analyses, eight suggested a benefit^40–46^ and two reported a neutral effect^30^ (*Table 2*).

### Beta-blockers

#### Heart failure with reduced ejection fraction

Several landmark RCTs demonstrated significant mortality benefit with the use of beta-blockers in HFrEF.^128–131^ Seventeen non-randomized studies reported 18 estimates of beta-blocker ‘treatment-effect’. Sixteen of these suggested beta-blocker use was associated with a lower mortality^28^^,^^30^^,^^46–58^ and two did not^30^^,^^59^ (*Table 3*).

#### Heart failure with preserved ejection fraction

The effect of beta-blockers on mortality was examined in one small randomized trial^136^ and a pre-specified subgroup analysis of a randomized trial which included patients with both HFrEF and HFpEF.^132^ Overall, we identified 13 non-randomized studies of beta-blockers in HFpEF, of which nine reported an association between beta-blocker use and better survival,^32^^,^^46^^,^^50^^,^^51^^,^^55^^,^^60–63^ whereas four did not^30^^,^^53^^,^^64^ (*Table 3*).

#### Mixed/unspecified heart failure phenotype

One moderately large RCT evaluated the effects of nebivolol in patients with both HFrEF and HFpEF, demonstrating a neutral effect on mortality.^137^ We identified 17 observational studies reporting 19 estimates of the ‘effect’ of treatment, with 17 suggesting benefit,^41^^,^^44–46^^,^^55^^,^^65–74^ and two reporting no difference in outcome between those treated with and not treated with a beta-blocker^30^ (*Table 3*).

### Mineralocorticoid receptor antagonists

#### Heart failure with reduced ejection fraction

Two pivotal RCTs in HFrEF demonstrated the mortality and hospitalization benefits of MRAs.^138^^,^^139^ In contrast, of 12 non-randomized studies only one concluded MRAs were of benefit,^75^ with 10 finding a neutral effect,^30^^,^^54^^,^^76–82^ and one suggesting a harmful effect^83^ (*Table 4*).

#### Heart failure with preserved ejection fraction

One large RCT showed no effect of spironolactone on mortality in patients with HFpEF.^141^ Two observational studies also found a neutral effect,^30^^,^^85^ but a further non-randomized study reported an association between MRA use and lower mortality^84^ (*Table 4*).

#### Mixed/unspecified heart failure phenotype

Of five studies of patients with a mixed HF phenotype, two suggested benefit,^84^^,^^86^ and three reported a neutral effect^30^^,^^46^ (*Table 4*).

### Statins

#### Heart failure with reduced ejection fraction

Two large RCTs showed a neutral effect of rosuvastatin on mortality in HFrEF (one trial included a small number of patients with HFpEF).^142^^,^^144^ Sixteen non-randomized studies reported 17 estimates of the ‘effect’ of statin treatment in HFrEF. Of these, 14 reported an association between statin use and better outcome,^28^^,^^59^^,^^87–97^ whereas only three found no association^30^^,^^59^^,^^98^ (*Table 5*).

#### Heart failure with preserved ejection fraction

The use of statins has not been evaluated in a randomized trial in patients with HFpEF, therefore, no relevant non-randomized studies were identified.

#### Mixed/unspecified heart failure phenotype

One large statin trial included patients with both HFrEF and HFpEF and showed no effect of treatment on mortality.^144^ Eleven observational studies reported 12 estimates of the ‘effect’ of a statin in patients with a mixture of HFrEF and HFpEF phenotypes, or where EF was not specified. Of these, 11 reported an association between statin use and better outcome,^45^^,^^68^^,^^88^^,^^98–104^ with only one describing no relationship between treatment and mortality^30^ (*Table 5*).

### Digoxin

#### Heart failure with reduced ejection fraction

A single RCT, the Digitalis Investigators Group (DIG) trial, showed that, in sinus rhythm, digoxin had a neutral effect on death but reduced the risk of HF hospitalization.^145^ Nine non-randomized studies reported 10 estimates of the ‘effect’ of digoxin treatment in HFrEF, with five concluding digoxin was harmful,^107–110^ four reporting a neutral effect,^30^^,^^55^^,^^106^ and one suggesting digoxin was beneficial^105^ (*Table 6*).

#### Heart failure with preserved ejection fraction

A single randomized trial of modest size, the DIG ancillary trial in HFpEF (*n* = 988), showed no effect of digoxin on mortality in patients with HFpEF in sinus rhythm, although the estimate of the effect of treatment was not robust because of limited power.^146^ Four observational studies were identified, one suggesting that non-randomized digoxin treatment was beneficial,^105^ and three showing a neutral association between treatment and mortality^30^^,^^55^ (*Table 6*).

#### Mixed/unspecified heart failure phenotype

The combined main and ancillary DIG trials showed a neutral effect of digoxin on mortality.^147^ Fourteen observational studies reported effect estimates for digoxin in patients with HFrEF and HFpEF in combination, or where EF was not specified. These studies reported 16 estimates of ‘treatment-effect’. Seven found an association between the use of digoxin and a higher mortality,^41^^,^^65^^,^^113–117^ seven were neutral,^30^^,^^42^^,^^55^^,^^112^^,^^113^ and two suggested better outcomes associated with digoxin use^105^^,^^111^ (*Table 6*).

## Discussion

There is a particularly strong evidence base for the treatment of HF, making it an appropriate condition in which to compare treatment effects established in RCTs with those estimated in non-randomized and observational studies.

Looking first at patients with HFrEF, six observational studies (reporting seven ‘effect’ estimates) fulfilled our inclusion criteria, and examined the association between treatment with an ACEI/ARB and mortality. Of these, five showed a lower mortality in patients receiving treatment of this type,^26–29^ whereas two did not,^30^ i.e. there was relatively good concordance between these non-randomized studies and the pivotal RCTs. However, the same concordance was not found in studies in HFpEF (see below).

The non-randomized analyses of beta-blockers in HFrEF also showed good agreement with the RCTs, with 16 of 18 analyses concordant.^28^^,^^30^^,^^46–59^ However, this was not the case in observational studies of patients with a mixed HF phenotype, where the Study of the Effects of Nebivolol Intervention on Outcomes and Rehospitalisation in Seniors with Heart Failure (SENIORS) trial had shown a neutral effect on mortality.^137^ Of the 19 non-randomized analyses, 17 showed a lower mortality among patients of this type treated with a beta-blocker.^30^^,^^41^^,^^44–46^^,^^55^^,^^65–74^

However, the picture was quite different for MRAs, which reduce mortality in HFrEF. Of 12 observational studies, one reported lower mortality in patients treated with a MRA,^75^ 10 did not find a better or worse outcome (i.e. were neutral),^30^^,^^54^^,^^76–82^ and one found a higher mortality (worse outcome) in the MRA treated patients.^83^ It is worth exploring this discordance in more detail. By far the largest study included 18 852 patients from Sweden and is worth examining in detail.^79^ The authors of this study used matching of spironolactone treated (*n* = 6551) and untreated (*n* = 12 301) patients. The authors also attempted to adjust for residual confounding in several different ways. Despite these statistical approaches, the multivariate HR for all-cause mortality with spironolactone vs. no spironolactone was 1.05 [95% confidence interval (CI) 1.00–1.11; *P* = 0.054] in the model adjusted for propensity score and 1.10 (95% CI 1.02–1.19; *P* = 0.020) in a 1:1 matched model. These findings stand in stark contrast to two separate trials of MRAs in HFrEF. The authors of the above observational study argued that the severity of HF symptoms and concomitant use of beta-blockers might explain the difference between their findings and the Randomized Aldactone Evaluation Study (RALES) trial, which used spironolactone in severely symptomatic patients, few of which were treated with a beta-blocker.^139^ However, patients with mild symptoms, the large majority of which were treated with a beta-blocker, were enrolled in the Eplerenone in Mild Patients Hospitalization And Survival Heart Failure (EMPHASIS-HF) trial, which demonstrated a clear mortality benefit of the MRA eplerenone.^138^ As an alternative explanation for their discrepant findings, the authors postulated that trial inclusion/exclusion criteria select patients more likely to benefit and less likely to experience harm pointing out, for example, the younger average age of patients in RALES (65 years) compared with the Swedish registry (71 years); however, the average age in EMPHASIS-HF was 69 years. In any case (and counterintuitively), the authors own analysis showed a significant treatment-by-age interaction whereby older (rather than younger) patients did better with MRA treatment.^79^ Several other of the authors’ subgroup analyses (e.g. significantly better outcome with an MRA in patients without diabetes compared to with diabetes) are directly contradicted by independent but consistent subgroup analyses from RALES and EMPHASIS-HF. The authors of the Swedish study also speculated that patients in the ‘real-world’ treated with a MRA maybe at greater risk of harm because of less careful monitoring of renal function and potassium.

Another notable example of a discrepancy between observational data and randomized trials does address issues of safety and generalisability. All but three of a remarkable 17 observational ‘effect’ estimates suggested that statins have a mortality benefit in HFrEF,^28^^,^^30^^,^^59^^,^^87–98^ yet two large independent RCTs showed no effect of this type of treatment on death.^142^^,^^144^ In patients with the mixed/unspecified HF phenotype, a further 11 of 12 analyses reported an association of statin use with mortality benefit.^30^^,^^45^^,^^68^^,^^88^^,^^98–104^ Again, it is instructive to examine one of the observational studies in detail. Go *et al.*^104^ used a Kaiser Permanente dataset with almost 25 000 patients to conduct careful propensity score-adjusted analyses of outcome related to statin treatment; the authors also used time-varying covariate adjustment for statin initiation during follow-up. The adjusted HR for all-cause mortality in patients treated with a statin (compared with those who were not) was 0.66 (95% CI 0.61–0.71) in individuals with CHD and 0.60 (95% CI 0.54–0.67) in those without CHD. Apart from the improbably large ‘reduction’ in mortality (34–40%), the similar ‘effect’ in patients with and without CHD seems unlikely given everything we know about the actions of statins. Moreover, the prior arguments made about generalisability and safety would need to be inverted here as the observational datasets included broad populations of patients with HF, presumably, receiving less intense monitoring than in the clinical trials.

Even in HFpEF, there are clearly discrepant findings between a large observational dataset and two randomized trials with an ARB^123^^,^^124^ and one trial with an ACEI.^122^ Once again, the most obvious example involves the Swedish HF registry.^29^ As previously, the authors of this study used an age- and propensity score-matched cohort. The adjusted HR for all-cause mortality in patients treated with an ACEI or ARB, compared with those not treated with one of these agents, was 0.90 (95% CI 0.85–0.96; *P* = 0.001). The authors also described a ‘dose–response’ relationship whereby the HR for high-dose treatment compared with no treatment was 0.85 (95% CI 0.78–0.83) and compared with low-dose treatment was 0.94 (95% CI 0.87–1.02). For this study, the authors used the issue of generalisability to explain why they saw benefit compared with the prior trials, in contradistinction to the case for MRAs where the opposite argument was made. Specifically, in this case, with ACEIs and ARBs, they argued that the broader, older and higher-risk population in the registry responded favourably to treatment compared with the more selected participants enrolled in the trials.

Much has been written recently in relation to the safety of digoxin in atrial fibrillation. Indeed, in a very illustrative example of the unreliability of observational data, Bavendiek *et al.*^148^ highlighted how in three separate and independent *post hoc* analyses of the same dataset, digoxin treatment was variably associated with increased all-cause mortality, was not associated with increased mortality and, in the third analysis, was associated with decreased in mortality in patients with an EF less than 30%. In HF, there is the same type of discrepancy between observational data and the single large RCT in HFrEF, an ancillary trial in HFpEF, and the combined analysis of the effect of digoxin in both HF phenotypes.^145–147^ In each of these analyses, digoxin had a neutral effect on all-cause mortality. A total of 30 observational analyses variously show better, worse, and neutral outcomes.^30^^,^^41^^,^^42^^,^^55^^,^^65^^,^^105–117^

Why the non-randomized analyses of outcomes related to use of ACEI/ARB and beta-blockers in HFrEF were generally (but not absolutely) concordant with the RCTs, in contrast to the other treatments examined, is an interesting question. There may be less confounding by indication, i.e. ACEIs/ARBs and beta-blockers are recommended in essentially all patients with HFrEF, whereas digoxin and, at least until recently, MRAs were reserved for patients with more advanced HF. There may also have been particularly strong publication bias making it difficult to report studies suggesting that use of ACEIs/ARBs or beta-blockers is not associated with better outcomes (or even associated with worse outcomes). Of course, with both treatments there is also a strong selection bias whereby the sickest patients are least likely to be prescribed (and to tolerate) these therapies. The opposite consideration may apply to the non-randomized studies showing an association between treatments such as statins and lower mortality, with the possibility of other biases such as the ‘healthy-user effect’ not fully adjusted for.

Although our analyses show that the findings of non-randomized studies of the association between treatment use and outcomes are frequently inconsistent, they do not mean observational studies/registries are of no value. Registry-based analyses may be all that is available where randomized trials are not possible, such as in rare diseases or for rare outcomes. The latter forms the basis of pharmaco-epidemiological surveillance for rare adverse effects of drugs not identified in clinical trials. Non-randomized analyses may provide information on under-studied groups or subgroups excluded from clinical trials. However, the results of such analyses must be interpreted with caution, especially if the results of different analyses of this type conflict. Registries serve an important function in describing the use (or under-use) of evidence-based therapies in the ‘real-world’, often leading to initiatives to improve prescribing. Perhaps the greatest value of registries is the potential they offer to conduct more ‘real-world’ randomized trials, i.e. to randomize patients in a registry to treatment and follow their outcomes within the registry. This approach has been pioneered in a study of thrombus aspiration in ST-segment elevation myocardial infarction using the Swedish Coronary Angiography and Angioplasty Registry^149^ and a similar approach is now being used to conduct the Spironolactone Initiation Registry Randomized Interventional Trial in Heart Failure with Preserved Ejection Fraction (SPIRRIT-HFpEF)^150^ in the Swedish HF Registry [NCT02901184].

Our study has a number of strengths and limitations. The strengths include the robust evidence base in HF, with often more than one randomized trial supporting the use or avoidance of specific therapies. There is a specific limitation in relation to the effect of MRAs in HFpEF. In the single, prospective, RCT, ineligible patients were included, and study drug was not administered, at certain investigative sites.^141^ As a result, the integrity of the trial has been questioned, as has the overall treatment effect observed.^151^ Examination of the effect of therapy in regions where the trial is thought to have been conducted as intended suggested possible benefit of spironolactone, compared with placebo.^140^ Consequently, the effect of spironolactone in this RCT and in the one observational analysis which suggested no benefit from MRA therapy may not be in agreement.

## Conclusion

This comprehensive comparison of the robust evidence base in HF with an increasing number of non-randomized data shows that it is not possible to make reliable therapeutic inferences from observational associations. While trials undoubtedly leave gaps in evidence and enrol selected participants, they clearly remain the best guide to the treatment of patients.

**Conflict of interest:** P.S.J. reports having received consulting fees from Novartis, research funding from Boehringer Ingelheim and serving on an advisory board for Vifor Pharma, all outside the submitted work. J.J.V.M. reports payments for trial-related activities to the University of Glasgow from Novartis, Cardiorentis, Amgen, Oxford University/Bayer, GlaxoSmithKline, Theracos, Abbvie, DalCor, Pfizer, Merck, AstraZeneca, Bristol Myers Squibb, and Kidney Research UK (KRUK)/Kings College Hospital, London/Vifor-Fresenius Pharma, all outside the submitted work.

## Supplementary Material

Supplementary Data
