# Supplementary material for: Association is not causation: treatment effects cannot be estimated from observational data in heart failure
Source: Eur Heart J. 2018 Aug 1;39(37):3417–38. doi: 10.1093/eurheartj/ehy407 (PMC6166137; doi:10.1093/eurheartj/ehy407)
Supplement: Supplementary Data [file ehy407_suppl_data.zip › Supplementary - Figure 1 - Rush - Association not causation.docx]

**FIGURE 1: PRISMA FLOW DIAGRAM FOR ACEI/ARB HF STUDIES**

ACEI = angiotensin-converting enzyme inhibitor; ARB = angiotensin receptor blocker; HF = heart failure; PRISMA = Preferred Reporting Items for Systematic reviews and Meta-Analyses; RCTs = randomized controlled trials.

Included full-text articles
(n = 31)

RCTs: n = 10

Observational studies: n = 21

Reasons for exclusion:

- Case studies, animal studies or in vitro studies
- Duplication
- Insufficient population size
- No “effect” estimate for all-cause mortality reported
- No suitable comparator group
- Reviews, letters, congress abstracts or editorials
- Study population overlapping with that of another larger study

n = 31

n = 599

Full-text articles assessed for eligibility
(n = 62)

Records screened
(n = 661)

Records after duplicates removed
(n = 661)

Additional records identified through other sources
(n = 2)

Records identified through database searching
(n = 1081)
