# Supplementary material for: Association is not causation: treatment effects cannot be estimated from observational data in heart failure
Source: Eur Heart J. 2018 Aug 1;39(37):3417–38. doi: 10.1093/eurheartj/ehy407 (PMC6166137; doi:10.1093/eurheartj/ehy407)
Supplement: Supplementary Data [file ehy407_suppl_data.zip › Supplementary - Figure 3 - Rush - Association not causation.docx]

**FIGURE 3: PRISMA FLOW DIAGRAM FOR MRA HF STUDIES**

HF = heart failure; MRA = mineralocorticoid receptor antagonist; PRISMA = Preferred Reporting Items for Systematic reviews and Meta-Analyses; RCTs = randomized controlled trials.

Reasons for exclusion:

- Case studies, animal studies or in vitro studies
- Duplication
- Insufficient population size
- No “effect” estimate for all-cause mortality reported
- No suitable comparator group
- Reviews, letters, congress abstracts or editorials
- Study population overlapping with that of another larger study

Included full-text articles
(n = 19)

RCTs: n = 4

Observational studies: n = 15

n = 11

n = 332

Full-text articles assessed for eligibility
(n = 30)

Records screened
(n = 362)

Records after duplicates removed
(n = 362)

Additional records identified through other sources
(n = 6)

Records identified through database searching
(n = 584)
