# Supplementary material for: Association is not causation: treatment effects cannot be estimated from observational data in heart failure
Source: Eur Heart J. 2018 Aug 1;39(37):3417–38. doi: 10.1093/eurheartj/ehy407 (PMC6166137; doi:10.1093/eurheartj/ehy407)
Supplement: Supplementary Data [file ehy407_suppl_data.zip › Supplementary - Table 1 - Rush - Association not causation.docx]

**Table 1: Quality assessment of randomized controlled trials (Cochrane Collaboration Risk of Bias tool for randomized controlled trials)**

| **Study** | **Sequence generation** | **Allocation concealment** | **Blinding of personnel** | **Blinding of outcome** | **Incomplete outcome data** | **Selective reporting** | **Other threats to validity** | **Overall rating** |
| --- | --- | --- | --- | --- | --- | --- | --- | --- |
| Ahmed, 2006 (DIG Ancillary Trial) | Low | Low | Low | Low | Low | Low | Unclear | Good |
| ANZ HF Research Collaborative Group, 1997 (ANZ) | Low | Low | Low | Low | Low | Low | High | Fair |
| BEST Investigators, 2001 (BEST) | Low | Low | Low | Low | Low | Low | Unclear | Good |
| CIBIS Investigators, 1994 (CIBIS-I) | Low | Low | Low | Low | Low | Low | High | Fair |
| CIBIS Investigators, 1999 (CIBIS-II) | Low | Low | Low | Low | Low | Low | Low | Good |
| Cleland, 2006 (PEP-CHF) | Low | Low | Low | Low | Low | Low | High | Fair |
| Cohn, 1991 (V-HeFT-II) | Low | Low | Low | Low | Low | Low | Low | Good |
| CONSENSUS Trial Study Group, 1987 (CONSENSUS) | Low | Low | Low | Low | Low | Low | High | Fair |
| DIG Investigators, 1997 (DIG Main Trial) | Low | Low | Low | Low | Low | Low | Low | Good |
| Flather, 2005 (SENIORS) | Low | Low | Low | Low | Low | Low | Low | Good |
| Granger, 2003 (CHARM-Alternative) | Low | Low | Low | Low | Low | Low | Low | Good |
| Jong, 2003 (X-SOLVD) | Low | Low | Low | Low | Low | Low | Low | Good |
| Kjekshus, 2007 (CORONA) | Low | Low | Low | Low | Low | Low | Low | Good |
| Massie, 2008 (I-PRESERVE) | Low | Low | Low | Low | Low | Low | Unclear | Good |
| MERIT-HF Study Group, 1999 (MERIT-HF) | Low | Low | Low | Low | Low | Low | Low | Good |
| Packer, 1996 (US Carvedilol HF Study Group) | Low | Low | Low | Low | Low | Low | Low | Good |
| Packer, 2001 (COPERNICUS) | Low | Low | Low | Low | Low | Low | Low | Good |
| Pfeffer, 2003 (CHARM Overall Programme) | Low | Low | Low | Low | Low | Low | Unclear | Good |
| Pfeffer, 2015 (TOPCAT - Americas, Russia/Georgia) | Low | Low | Low | Low | Low | High | High | Poor |
| Pitt, 1999 (RALES) | Low | Low | Low | Low | Low | Low | Low | Good |
| Pitt, 2014 (TOPCAT) | Low | Low | Low | Low | Low | Low | High | Fair |
| Rich, 2001 (DIG Overall) | Low | Low | Low | Low | Low | Low | Low | Good |
| SOLVD Investigators, 1991 (SOLVD-Treatment) | Low | Low | Low | Low | Low | Low | Low | Good |
| SOLVD Investigators, 1992 (SOLVD-Prevention) | Low | Low | Low | Low | Low | Low | Low | Good |
| Takano, 2013 (PEARL) | Low | Low | High | Low | Low | Low | Unclear | Poor |
| Tavazzi, 2008 (GISSI-HF Rosuvastatin) | Low | Low | Low | Low | Low | Low | Low | Good |
| van Veldhuisen, 2009 (SENIORS) | High | Low | Low | Low | Low | Low | High | Poor |
| Yamamoto, 2013 (J-DHF) | Unclear | Unclear | High | Low | Low | Low | High | Poor |
| Yusuf, 2003 (CHARM-Preserved) | Low | Low | Low | Low | Low | Low | Unclear | Good |
| Zannad, 2011 (EMPHASIS-HF) | Low | Low | Low | Low | Low | Low | Low | Good |
