# Supplementary material for: Association is not causation: treatment effects cannot be estimated from observational data in heart failure
Source: Eur Heart J. 2018 Aug 1;39(37):3417–38. doi: 10.1093/eurheartj/ehy407 (PMC6166137; doi:10.1093/eurheartj/ehy407)
Supplement: Supplementary Data [file ehy407_suppl_data.zip › Supplementary - Table 2 - Rush - Association not causation.docx]

**Table 2: Quality assessment of observational studies (Risk of Bias Assessment tool for Non-randomized Studies – RoBANS)**

| **Study** | **Selection of participants** | **Confounding variables** | **Measurement of exposure** | **Blinding of outcome** | **Incomplete outcome data** | **Selective reporting** | **Overall rating** |
| --- | --- | --- | --- | --- | --- | --- | --- |
| Ahmed, 2003 (Medicare) | High | Unclear | Unclear | Low | Unclear | Unclear | High |
| Ahmed, 2013 (Alabama HF Project) | High | Low | Low | Low | Unclear | Unclear | High |
| Ahmed, 2014 (Alabama HF Project) | High | Low | Low | Low | Unclear | Unclear | High |
| Alehagen, 2015 (Swedish HF Registry) | Low | Low | Low | Low | Low | Unclear | Low |
| Al-Khateeb, 2017 | Unclear | Low | Low | Low | Unclear | Unclear | Unclear |
| Andrey, 2011 | Low | Low | Low | Low | Unclear | Low | Low |
| Anker, 2006 (ELITE-II) | Low | Low | Low | Low | Low | Low | Low |
| Anker, 2006 (European Centres Study) | Low | Low | Low | Low | Unclear | Unclear | Low |
| Ballo, 2016 | High | Low | Low | Low | Low | Unclear | High |
| Bhatia, 2015 (Alabama HF Project) | High | Low | Low | Low | Unclear | Unclear | High |
| Bobbio, 2003 (BRING-UP) | High | Unclear | Low | Low | Low | Unclear | High |
| Bonsu, 2017 | Low | Low | Low | Low | Unclear | Unclear | Low |
| Bonsu, 2017 (statin) | Low | Low | Low | Low | Unclear | Unclear | Low |
| Butler, 2010 (Val-HeFT) | Low | Low | Low | Low | Low | Low | Low |
| Cadrin-Tourigny, 2017 | High | Low | Low | Low | Low | Unclear | High |
| Chan, 2005 (CHS) | Low | Low | Low | Low | Unclear | Unclear | Low |
| Coleman, 2008 | High | Low | Low | Low | Unclear | Low | High |
| Dauterman, 2001 (Medicare) | High | Unclear | Unclear | Low | Low | Unclear | High |
| Del Carlo, 2014 | High | Unclear | Unclear | Low | High | Unclear | High |
| Dhaliwal, 2008 | High | High | Unclear | Low | Unclear | Unclear | High |
| Dickinson, 2007 (SCD-HeFT) | High | Low | Low | Low | Low | Unclear | High |
| Dobre, 2007 | High | Low | Low | Low | Unclear | Unclear | High |
| Domanski, 2005 (SOLVD) | Low | Low | Low | Low | Low | Low | Low |
| Eisen, 2017 (ENGAGE AF-TIMI 48) | Low | Low | Low | Low | Low | Unclear | Low |
| El-Refai, 2013 | High | Low | Unclear | Low | Unclear | Unclear | High |
| Fauchier, 2009 | High | High | Unclear | Low | High | Unclear | High |
| Flory, 2012 (THIN) | Low | Low | Low | Low | Unclear | Low | Low |
| Foody, 2006 (NHC) | High | Low | Unclear | Low | Unclear | Unclear | High |
| Frankenstein, 2013 (Norwegian HF Registry) | Low | Low | Low | Low | Low | Unclear | Low |
| Freeman, 2013 (KPNC) | High | Low | Unclear | Low | Unclear | Unclear | High |
| Gastelurrutia, 2012 | Low | Low | Low | Low | Unclear | Unclear | Low |
| Go, 2006 (KPNC) | High | Low | Unclear | Low | Unclear | Unclear | High |
| Goldeberger, 2006 (DEFINITE) | High | Unclear | Low | Low | Low | Unclear | High |
| Gomez-Soto, 2010 (ACEI) | Low | Low | Low | Low | Low | Unclear | Low |
| Gomez-Soto, 2010 (statin) | Low | Low | Low | Low | Low | Unclear | Low |
| Gomez-Soto, 2011 (BB) | Low | Low | Low | Low | Low | Unclear | Low |
| Grigorian Shamagian, 2006 | Low | Unclear | Low | Low | Low | Unclear | Low |
| Hallberg, 2007 (RIKS-HIA) | High | Low | Unclear | Low | Low | Unclear | High |
| Hamaguchi, 2010 (JCARE-CARD) | Low | Low | Low | Low | Unclear | Unclear | Low |
| Hernandez, 2009 (OPTIMIZE-HF) | High | Low | Low | Low | Unclear | Unclear | High |
| Hernandez, 2012 (GWTG-Medicare) | High | Low | Low | Low | Unclear | Unclear | High |
| Huan Loh, 2007 | Low | Low | Low | Low | Unclear | Unclear | Low |
| Jordán, 2009 (BADAPIC) | Unclear | Low | Unclear | Low | Low | Unclear | Unclear |
| Jost, 2005 (Ludwigshafen HF Registry) | Low | Low | Low | Low | Unclear | Unclear | Low |
| Katz, 2016 | Low | Low | Low | Low | Unclear | Unclear | Low |
| Keyhan, 2007 (ACEI) | High | Low | Unclear | Low | Unclear | Unclear | High |
| Keyhan, 2007 (BB) | High | Low | Low | Low | Unclear | Unclear | High |
| Krum, 2007 (CIBIS-II) | Low | Low | Low | Low | Low | Low | Low |
| Krum, 2007 (Val-HeFT) | Low | Low | Low | Low | Low | Low | Low |
| Lam, 2017 (Alabama HF Project) | High | Low | Low | Low | Unclear | Unclear | High |
| Lee, 2013 (KPNC) | High | Low | Unclear | Low | Unclear | Unclear | High |
| Liu, 2014 | Low | Low | Low | Low | Low | Unclear | Low |
| Lund, 2012 (Swedish HF Registry) | Low | Low | Low | Low | Low | Unclear | Low |
| Lund, 2013 (Swedish HF Registry) | Low | Low | Low | Low | Low | Unclear | Low |
| Lund, 2014 (Swedish HF Registry) | Low | Low | Low | Low | Low | Unclear | Low |
| Madelaire, 2016 | High | Low | Low | Low | Low | Low | High |
| Maggioni, 2003 (BRING-UP) | High | High | Low | Low | Unclear | Unclear | High |
| Maison, 2012 | High | Low | Unclear | Low | Unclear | Unclear | High |
| Marijon, 2010 (EVADEF) | High | High | Unclear | Low | Low | Unclear | High |
| Masoudi, 2004 (NHC) | High | Unclear | Unclear | Low | Unclear | Unclear | High |
| McAlister, 1999 | Low | Unclear | Low | Low | Low | Unclear | Low |
| McCullough, 2003 (REACH) | High | Unclear | Unclear | Low | Unclear | Unclear | High |
| Miyagishima, 2009 | Low | Low | Low | Low | Unclear | Low | Low |
| Mozafarrian, 2004 (PRAISE) | Low | Low | Low | Low | Low | Unclear | Low |
| Mujib, 2013 (OPTIMIZE-HF) | High | Low | Low | Low | Unclear | Unclear | High |
| Nevzorov, 2009 | High | Low | High | Low | Unclear | Unclear | High |
| O'Meara, 2012 (AF-CHF) | High | Low | Low | Low | Low | Unclear | High |
| Ouzounian, 2007 (ICONS) | Unclear | Unclear | Low | Low | Unclear | Unclear | Unclear |
| Ouzounian, 2009 (EFFECT) | Low | Low | Low | Low | Unclear | Unclear | Low |
| Pascual-Figal, 2008 (BB) | Low | Low | Low | Low | Unclear | Unclear | Low |
| Pascual-Figal, 2013 (MUSIC) | Low | Low | Low | Low | Unclear | Unclear | Low |
| Patel, 2012 (OPTIMIZE-HF) | High | Low | Low | Low | Unclear | Unclear | High |
| Patel, 2013 (OPTIMIZE-HF) | High | Low | Low | Low | Unclear | Unclear | High |
| Patel, 2014 (OPTIMIZE-HF) | High | Low | Low | Low | Unclear | Unclear | High |
| Pedone, 2004 (GIFA) | Unclear | High | Low | Low | High | Unclear | High |
| Philbin, 1997 (MIS-CHF) | Low | Unclear | Low | Low | Unclear | Unclear | Unclear |
| Philbin, 2000 (MIS-CHF) | Low | Unclear | Low | Low | Unclear | Unclear | Unclear |
| Ray, 2005 | High | Low | Unclear | Low | Unclear | Unclear | High |
| Ruiz, 2016 | Low | Low | Low | Low | Low | Low | Low |
| Ryan, 2009 (THIN) | Low | Low | Low | Low | Low | Low | Low |
| Sanam, 2016 (Alabama HF Project) | High | Low | Low | Low | Unclear | Unclear | High |
| Shah, 2008 (NHC) | High | Low | Unclear | Low | Unclear | Unclear | High |
| Shah, 2014 | High | Low | Unclear | Low | Unclear | Low | High |
| Sin, 2002 | High | Low | Unclear | Low | Unclear | Unclear | High |
| Sligl, 2004 | Low | Low | Low | Low | Low | Unclear | Low |
| Sumner, 2009 (COMPANION) | High | Low | Low | Low | Low | Unclear | High |
| Tandon, 2004 | Low | Low | Low | Low | Unclear | Unclear | Low |
| Teng, 2010 | High | Unclear | Low | Low | Unclear | Low | High |
| Tribouilloy, 2008 | Low | Low | Low | Low | Low | Unclear | Low |
| Ushigome, 2015 (CHART-1, CHART-2) | Low | Low | Low | Low | Unclear | Low | Low |
| Whitbeck, 2012 | Unclear | Low | Low | Low | Low | Low | Low |
| Xu, 2013 | Low | Unclear | Low | Low | Low | Unclear | Low |
