# Supplementary figures and images for: Association is not causation: treatment effects cannot be estimated from observational data in heart failure
Source: Eur Heart J. 2018 Aug 1;39(37):3417–38. doi: 10.1093/eurheartj/ehy407 (PMC6166137; doi:10.1093/eurheartj/ehy407)

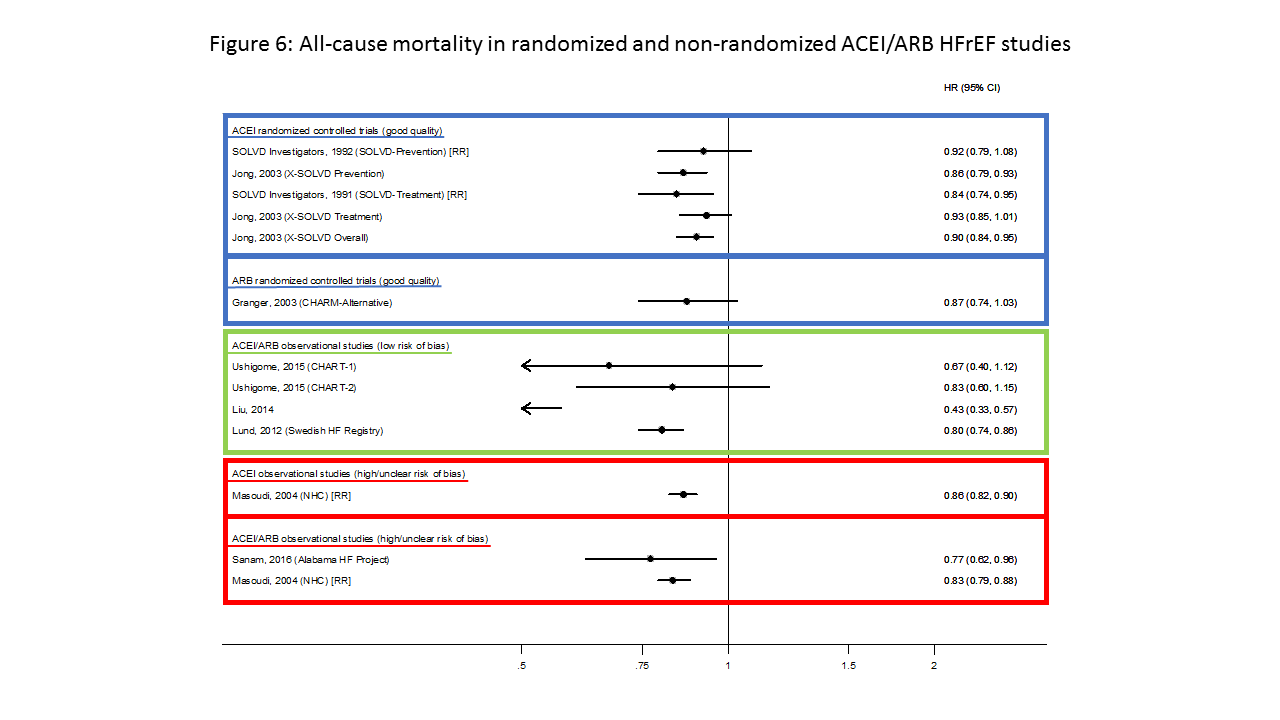

Supplement: Supplementary Data [file ehy407_suppl_data.zip › Supplementary - Figure 6 - Rush - Association not causation.tif]

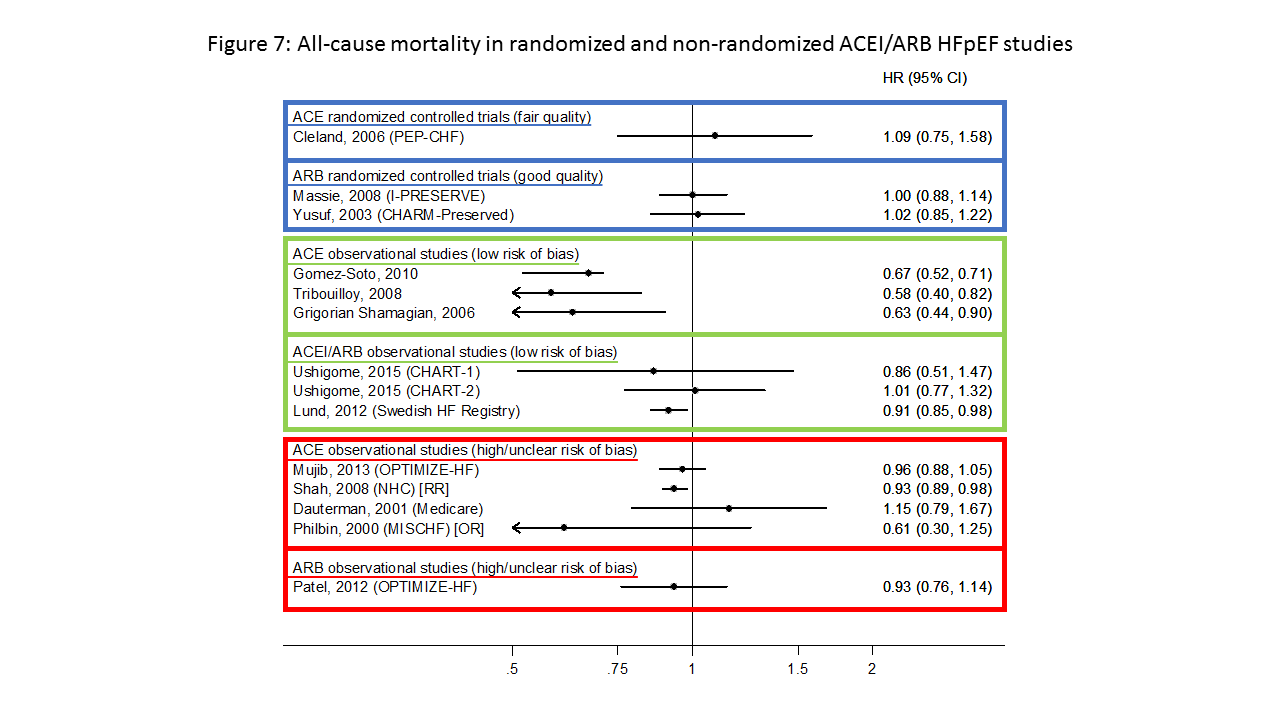

Supplement: Supplementary Data [file ehy407_suppl_data.zip › Supplementary - Figure 7 - Rush - Association not causation.tif]

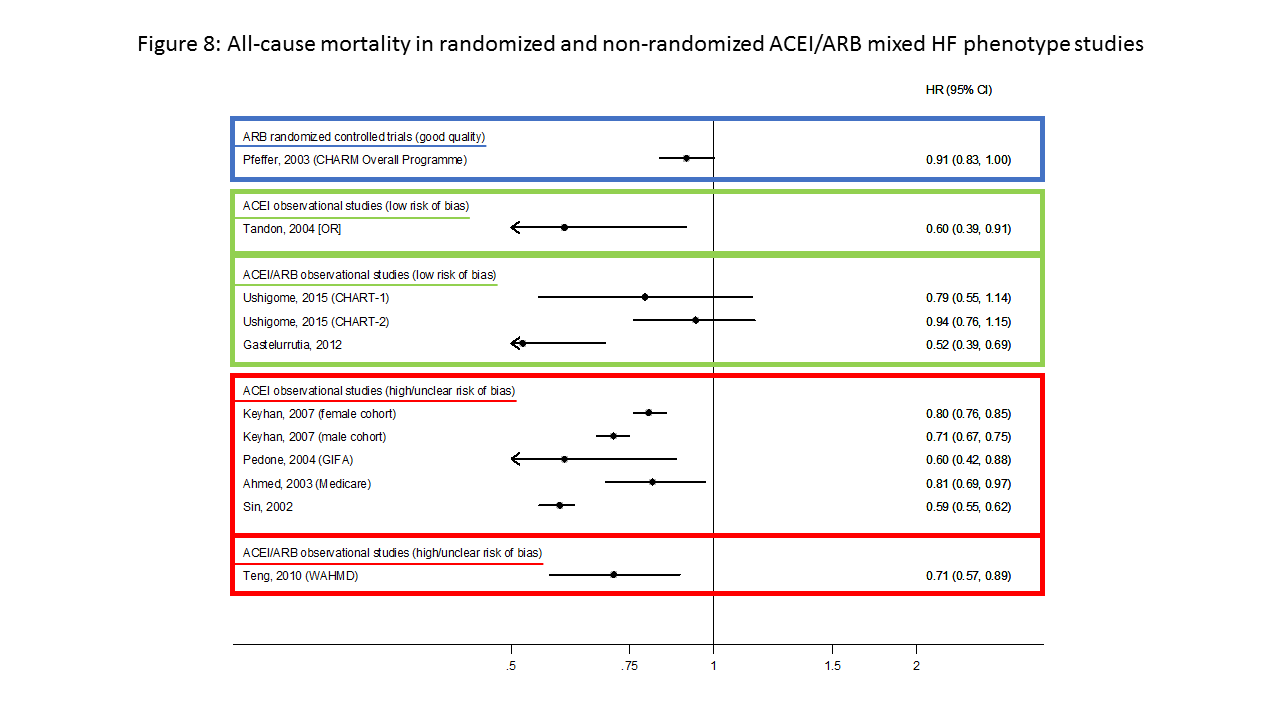

Supplement: Supplementary Data [file ehy407_suppl_data.zip › Supplementary - Figure 8 - Rush - Association not causation.tif]

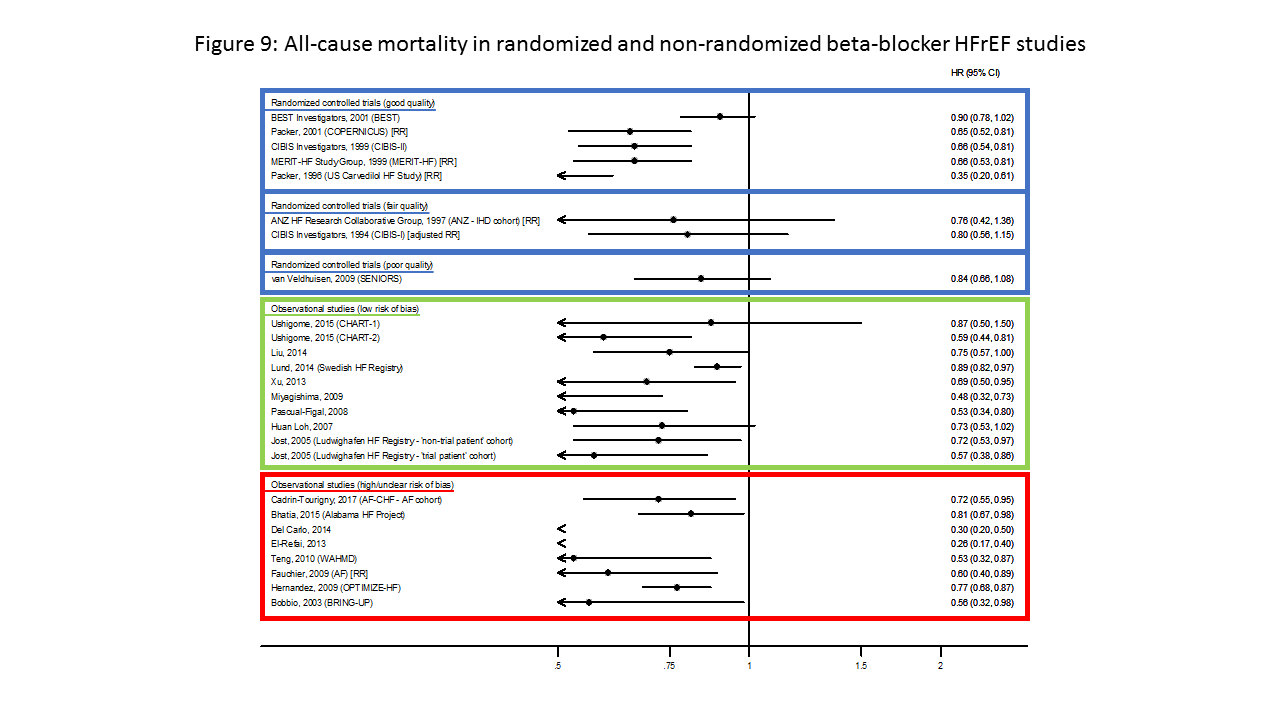

Supplement: Supplementary Data [file ehy407_suppl_data.zip › Supplementary - Figure 9 - Rush - Association not causation.tif]

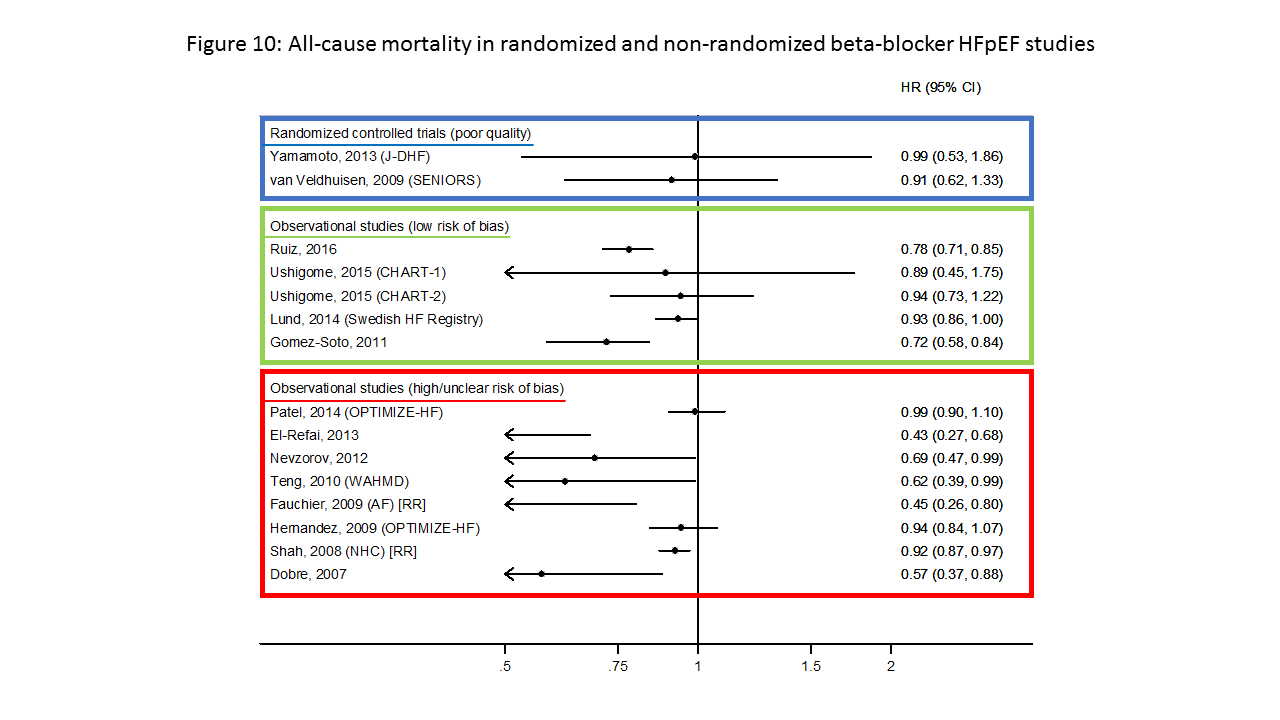

Supplement: Supplementary Data [file ehy407_suppl_data.zip › Supplementary - Figure 10 - Rush - Association not causation.tif]

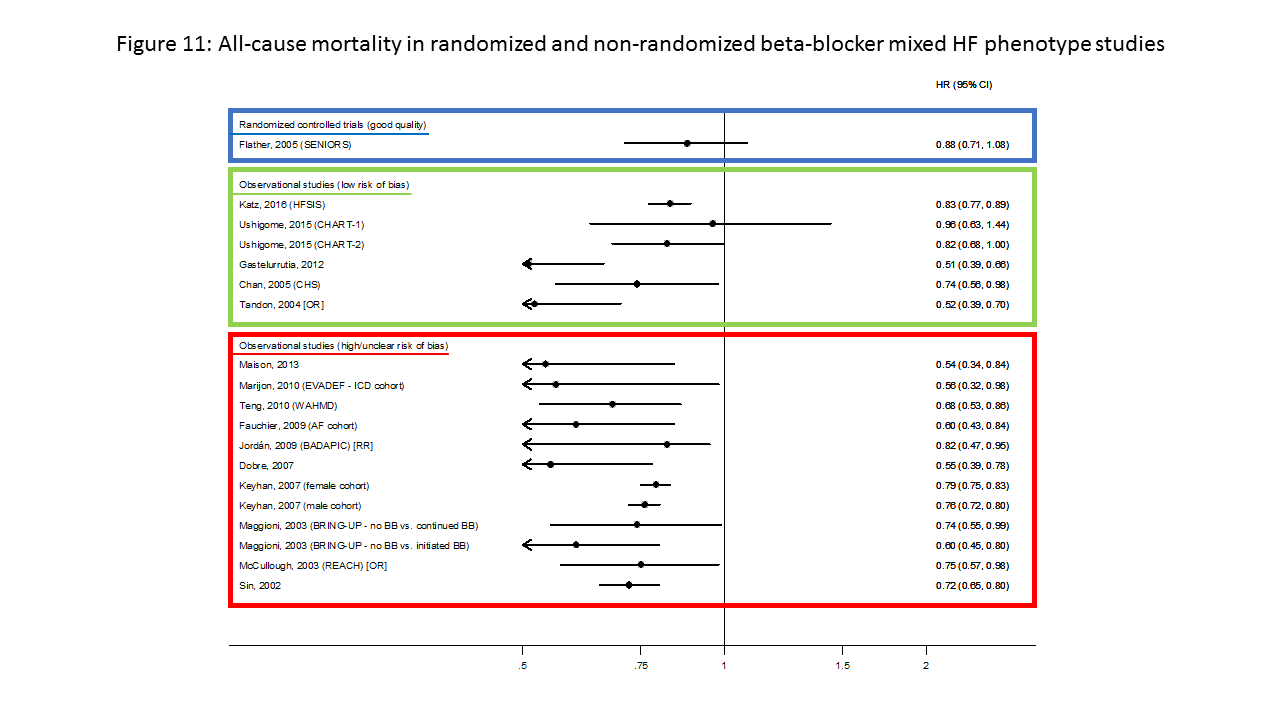

Supplement: Supplementary Data [file ehy407_suppl_data.zip › Supplementary - Figure 11 - Rush - Association not causation.tif]

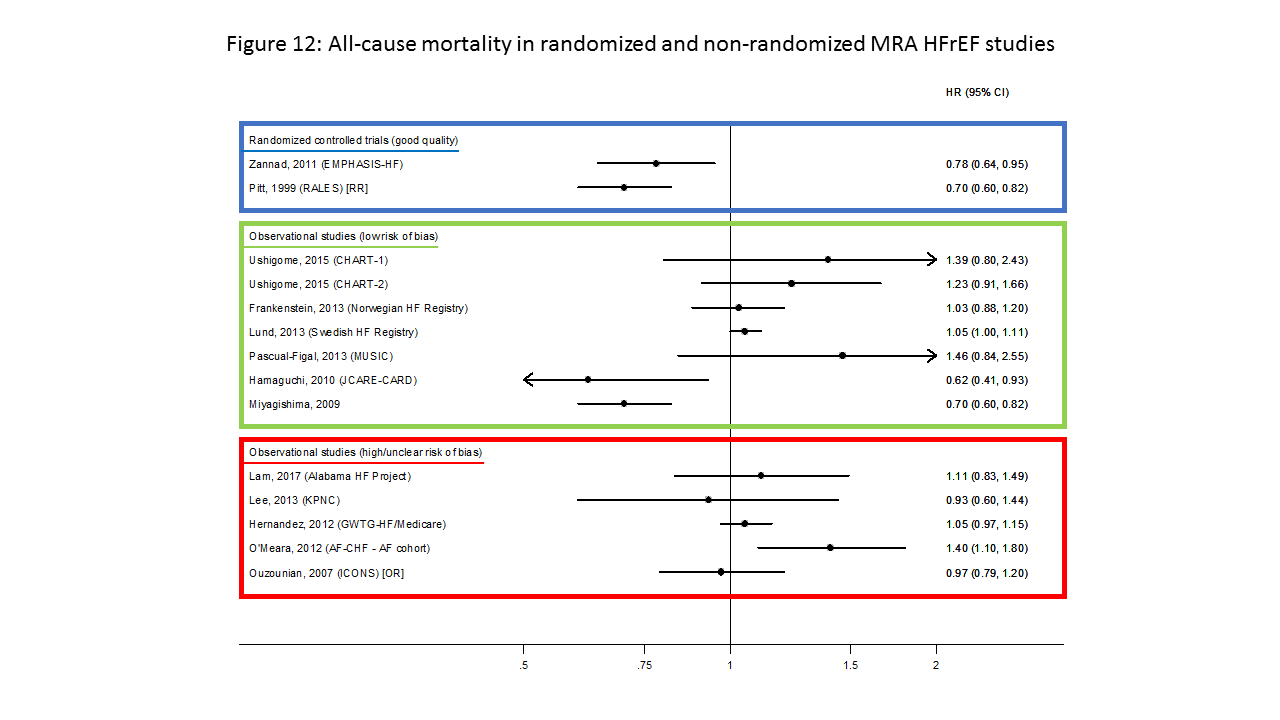

Supplement: Supplementary Data [file ehy407_suppl_data.zip › Supplementary - Figure 12 - Rush - Association not causation.tif]

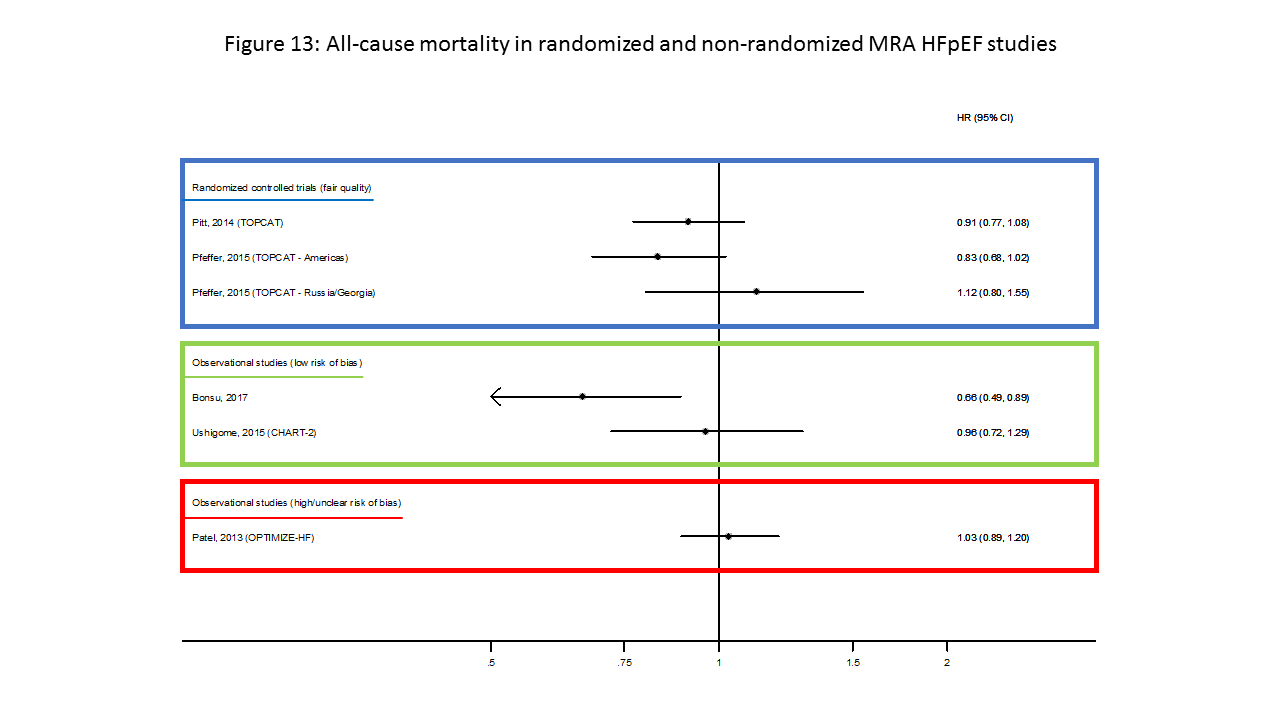

Supplement: Supplementary Data [file ehy407_suppl_data.zip › Supplementary - Figure 13 - Rush - Association not causation.tif]

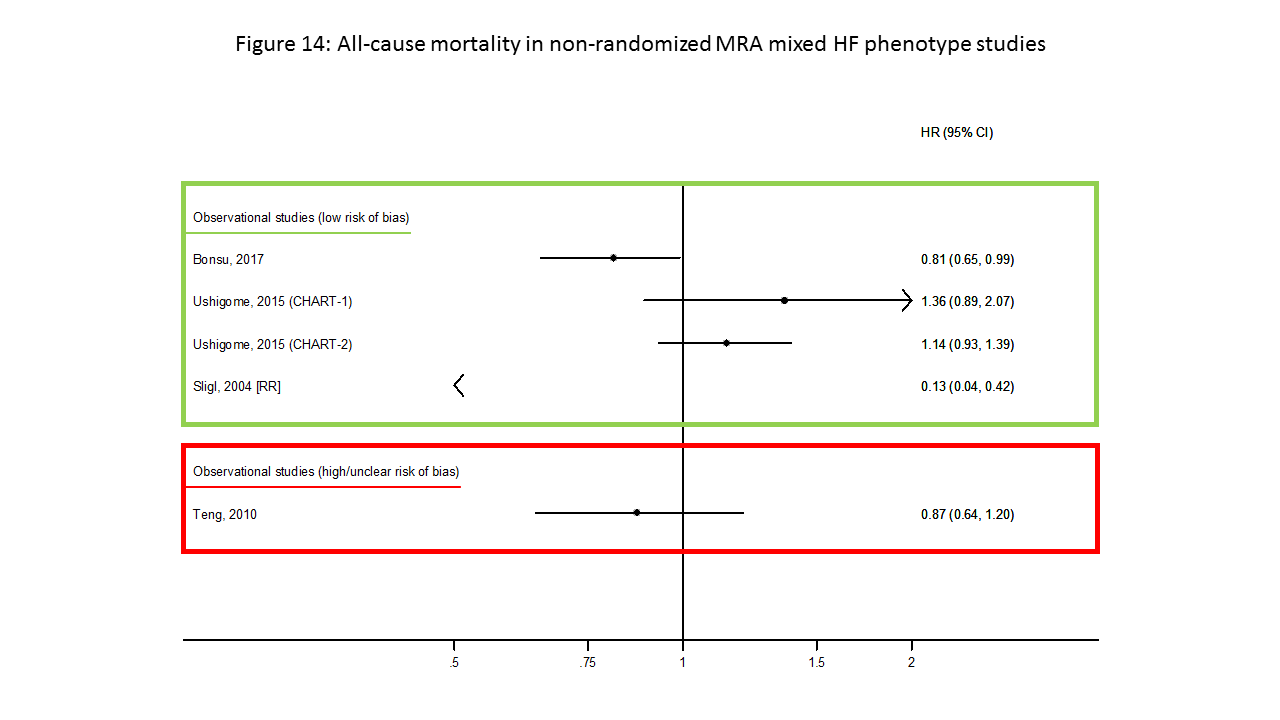

Supplement: Supplementary Data [file ehy407_suppl_data.zip › Supplementary - Figure 14 - Rush - Association not causation.tif]

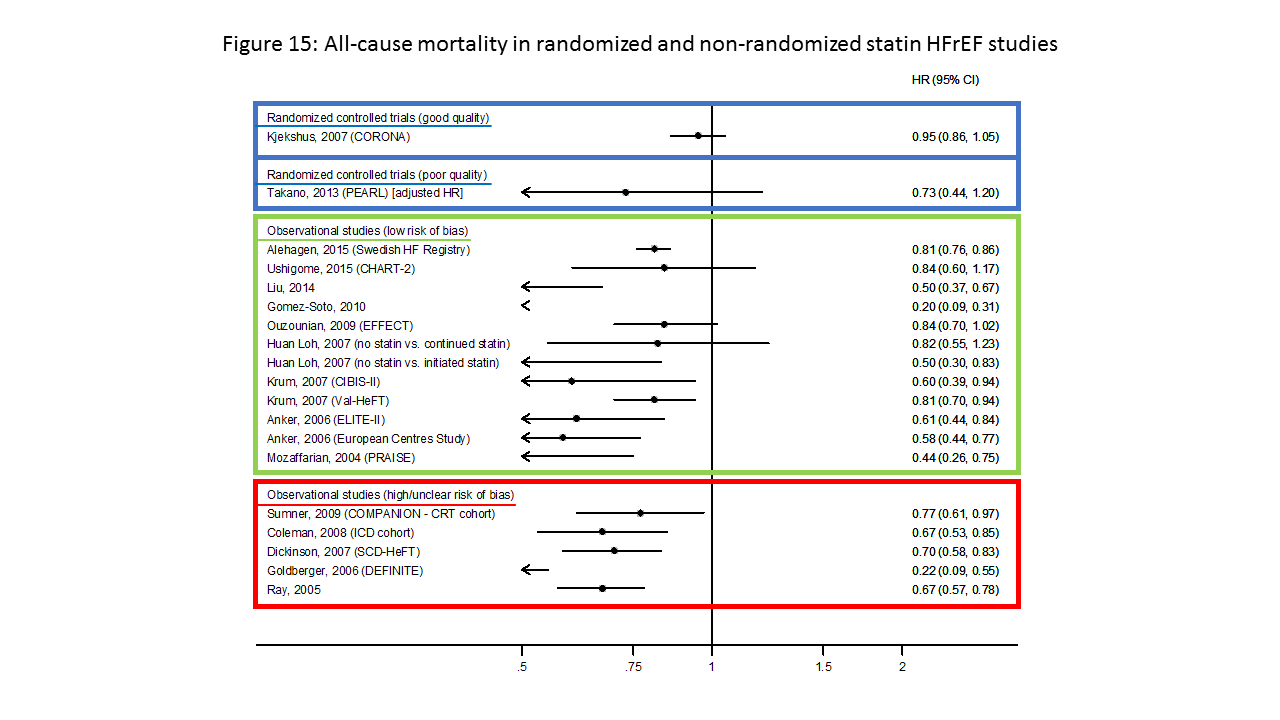

Supplement: Supplementary Data [file ehy407_suppl_data.zip › Supplementary - Figure 15 - Rush - Association not causation.tif]

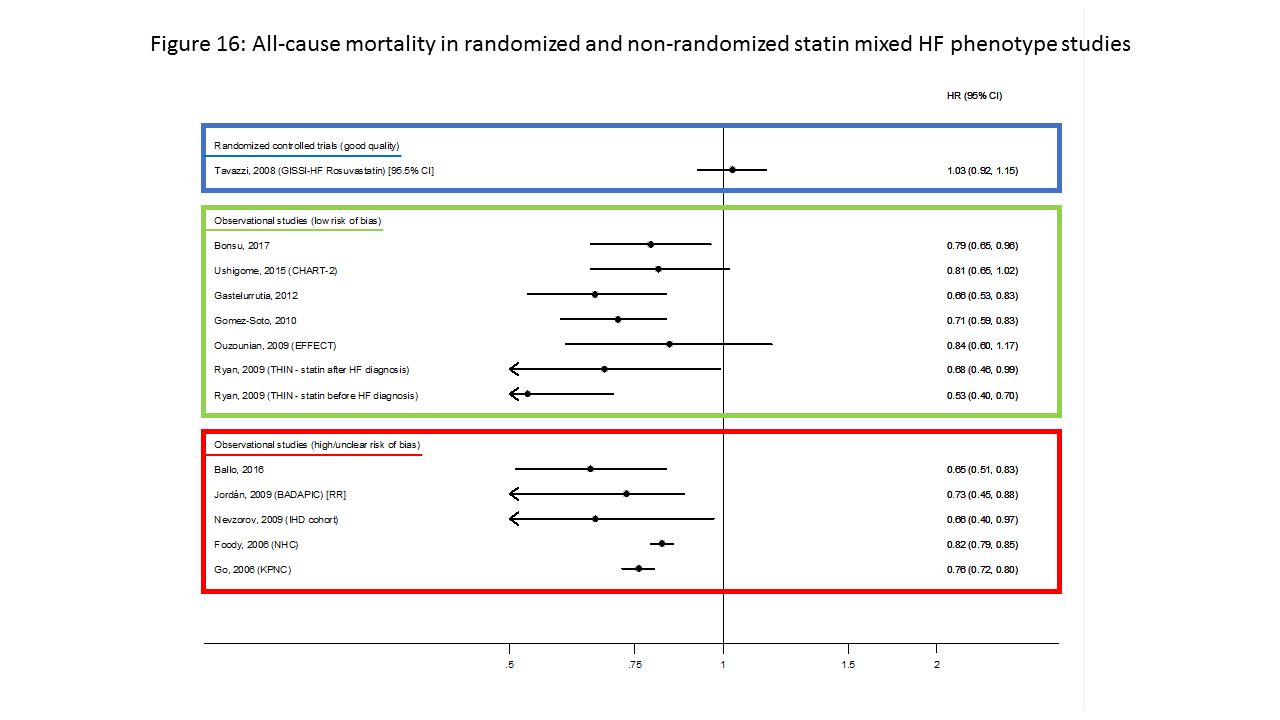

Supplement: Supplementary Data [file ehy407_suppl_data.zip › Supplementary - Figure 16 - Rush - Association not causation.tif]

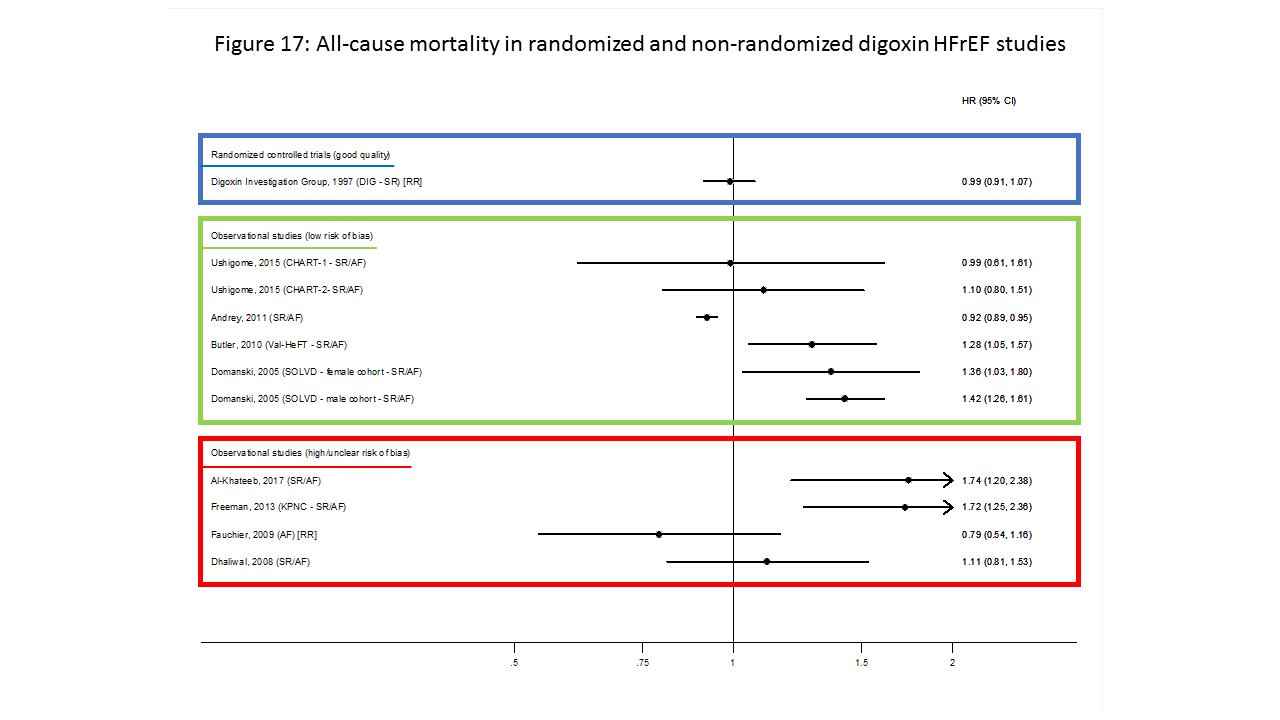

Supplement: Supplementary Data [file ehy407_suppl_data.zip › Supplementary - Figure 17 - Rush - Association not causation.tif]

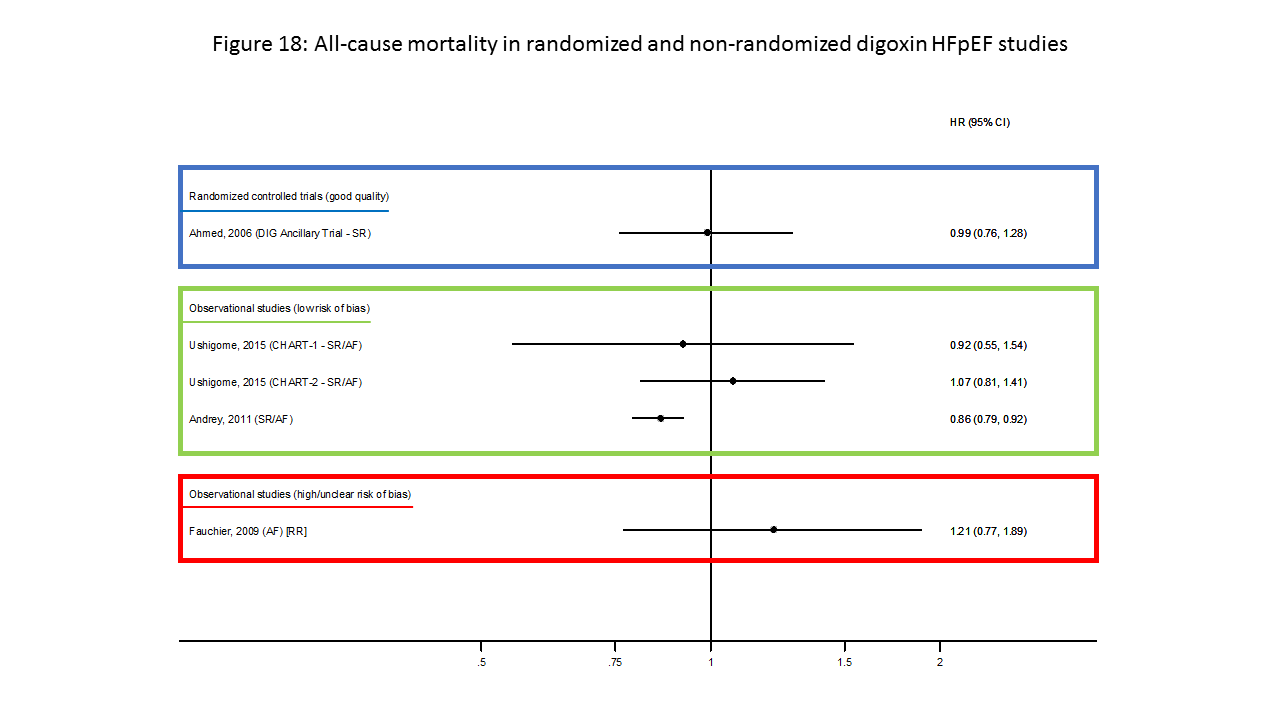

Supplement: Supplementary Data [file ehy407_suppl_data.zip › Supplementary - Figure 18 - Rush - Association not causation.tif]

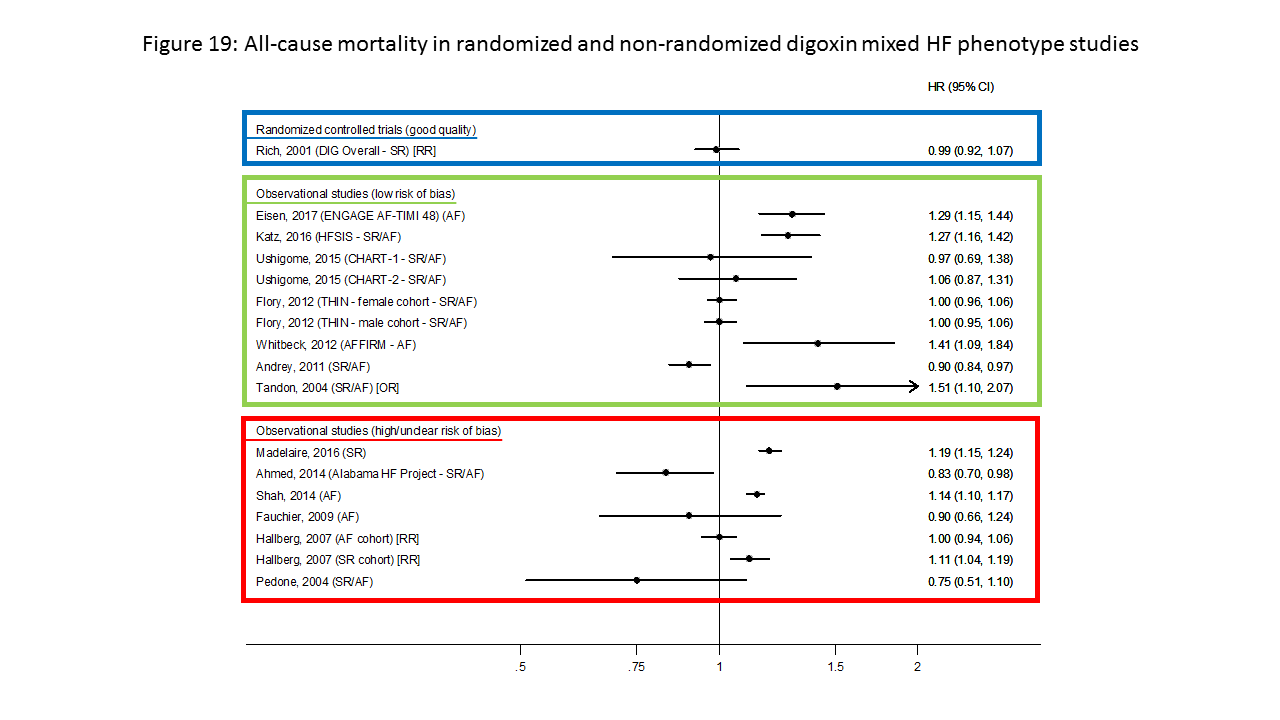

Supplement: Supplementary Data [file ehy407_suppl_data.zip › Supplementary - Figure 19 - Rush - Association not causation.tif]
